# Selective inhibition of HDAC6 regulates expression of the oncogenic driver EWSR1-FLI1 through the *EWSR1* promoter in Ewing sarcoma

**DOI:** 10.1038/s41388-021-01974-4

**Published:** 2021-08-03

**Authors:** Daniel J. García-Domínguez, Nabil Hajji, Sara Sánchez-Molina, Elisabet Figuerola-Bou, Rocío M. de Pablos, Ana M. Espinosa-Oliva, Eduardo Andrés-León, Laura Carmen Terrón-Camero, Rocío Flores-Campos, Guillem Pascual-Pasto, María José Robles, Isidro Machado, Antonio Llombart-Bosch, Giovanna Magagnoli, Katia Scotlandi, Ángel M. Carcaboso, Jaume Mora, Enrique de Álava, Lourdes Hontecillas-Prieto

**Affiliations:** 1 Institute of Biomedicine of Seville (IBiS), Hospital Universitario Virgen del Rocío/CSIC/University of Seville /CIBERONC, Seville, Spain; 2 Division of Brain Sciences, Imperial College London, London, United Kingdom; 3 Developmental Tumour Biology Laboratory, Hospital Sant Joan de Déu, Barcelona, Spain; 4 Department of Biochemistry and Molecular Biology, Faculty of Pharmacy, University of Seville, Seville, Spain; 5 Bioinformatics Unit, Instituto de Parasitología y Biomedicina “López-Neyra”, Consejo Superior de Investigaciones Científicas (IPBLN-CSIC), Granada, Spain; 6 Institut de Recerca Sant Joan de Deu, Pediatric Hematology and Oncology, Hospital Sant Joan de Deu, Barcelona, Spain; 7 Pathology Unit, Hospital Universitario Virgen del Rocío/CSIC/University of Seville/CIBERONC, Seville, Spain; 8 Pathology Department, Instituto Valenciano de Oncología, Valencia, Spain; 9 Pathology Department, University of Valencia, Valencia, Spain; 10 Department of Pathology, IRCCS Istituto Ortopedico Rizzoli, Bologna, Italy; 11 Experimental Oncology Laboratory, IRRCS Istituto Ortopedico Rizzoli, Bologna, Italy; 12 Department of Normal and Pathological Cytology and Histology, School of Medicine, University of Seville, Seville, Spain

**Keywords:** Oncogenes, Paediatric cancer

## Abstract

Ewing sarcoma (EWS) is an aggressive bone and soft tissue tumor of children and young adults in which the principal driver is a fusion gene, *EWSR1-FLI1*. Although the essential role of EWSR1-FLI1 protein in the regulation of oncogenesis, survival, and tumor progression processes has been described in-depth, little is known about the regulation of chimeric fusion-gene expression. Here, we demonstrate that the active nuclear HDAC6 in EWS modulates the acetylation status of specificity protein 1 (SP1), consequently regulating the SP1/P300 activator complex binding to *EWSR1* and *EWSR1-FLI1* promoters. Selective inhibition of HDAC6 impairs binding of the activator complex SP1/P300, thereby inducing *EWSR1-FLI1* downregulation and significantly reducing its oncogenic functions. In addition, sensitivity of EWS cell lines to HDAC6 inhibition is higher than other tumor or non-tumor cell lines. High expression of HDAC6 in primary EWS tumor samples from patients correlates with a poor prognosis in two independent series accounting 279 patients. Notably, a combination treatment of a selective HDAC6 and doxorubicin (a DNA damage agent used as a standard therapy of EWS patients) dramatically inhibits tumor growth in two EWS murine xenograft models. These results could lead to suitable and promising therapeutic alternatives for patients with EWS.

## Introduction

Ewing sarcoma (EWS) is a highly aggressive tumor that affects children and young adults [1], showing gene fusions involving one member of the FET family of genes (usually EWSR1) and a member of the ETS family of transcription factors, with *EWSR1-FLI1* being the most common [1]. Continuous expression of EWSR1-FLI1 in a permissive cellular context is essential both for preserving the malignant phenotype in EWS cells and for their survival, and its inhibition induces apoptosis [2–4]. Little is known about the molecular mechanisms regulating the expression of the chimeric fusion, although it is well known it is an aberrant transcription factor that can recruit transcription regulator proteins, such as lysine-specific histone demethylase 1A (LSD1) and histone deacetylases (HDACs) [5]. In addition, the chimeric protein regulates other essential mechanisms, thereby compromising the epigenetic homeostasis [6, 7], including deregulation of histone acetylation [8].

The involvement of HDACs in cancer boosted the development of several HDAC inhibitors for use in the clinic [9]. Recently, we have shown that the pan-HDAC inhibitor SAHA inhibits EWSR1-FLI1 expression [10]. However, it remains to be determined which HDAC(s) are involved specifically in the regulation of EWSR1-FLI1 expression. Usually, the treatment with non-selective HDAC inhibitors in patients is an imprecise mechanism associated with increased toxicity [11]. Hence, the identification of specific HDAC(s) involved in the regulation of EWSR1-FLI1, and the use of selective inhibitors, may improve efficacy and avoid toxicity of treatments, and enhance our understanding about the regulatory mechanisms of EWSR1-FLI1.

HDAC6, a class II HDAC, has not been specifically described in EWS. HDAC6 activity had been fundamentally observed in the cytoplasm [12], where α-tubulin and HSP90α have been defined as its substrates [13]. These proteins are involved in multiple cell functions, and deregulation of HDAC6 activity is associated with a variety of diseases, including cancer [12, 13]. Although HDAC6 localization is partially nuclear [14], it is not clear if it plays a functional role in gene regulation via the regulation of histone acetylation. In fact, several studies revealed a role of HDAC6 in regulating the activity of transcription factor modulators [15, 16]. Recently, we identified that HDAC6 specifically regulates histone acetylation at a specific residue, H4K12, in several types of cancer, including EWS [17]. From a clinical point of view, HDAC6 overexpression in many tumor types is associated with poor prognosis, drug resistance, cancer cell proliferation and migration [16, 18, 19]. Further, knocking down HDAC6 in mice has no effects on viability or fertility [20]. Overall, these results suggest HDAC6 being a safe target for EWS treatments.

As epigenetics plays a crucial role in EWS oncogenesis, we performed a screen assay of 43 epigenetic drugs in seven EWS cell lines to determine whether any of them prevents the oncogenic activities of EWSR1-FLI1. Our screen revealed that EWS cell lines were mostly sensitive to the specific HDAC6 inhibitor BML-281. We then aimed to investigate in-depth the role of HDAC6 in EWS cells in vivo and in vitro. This study presents first-time evidence that HDAC6 specifically regulates the expression of *EWSR1-FLI1* by modulating the binding of the SP1/P300 complex to the *EWSR1-FLI1* promoter region. Our results show the relevant role of HDAC6 in EWS and suggest the use of selective HDAC6 inhibitors as a novel therapeutic approach to treat this type of sarcoma.

## Results

### EWS cell lines are remarkably sensitive to selective inhibition of HDAC6

**Fig. 1:**
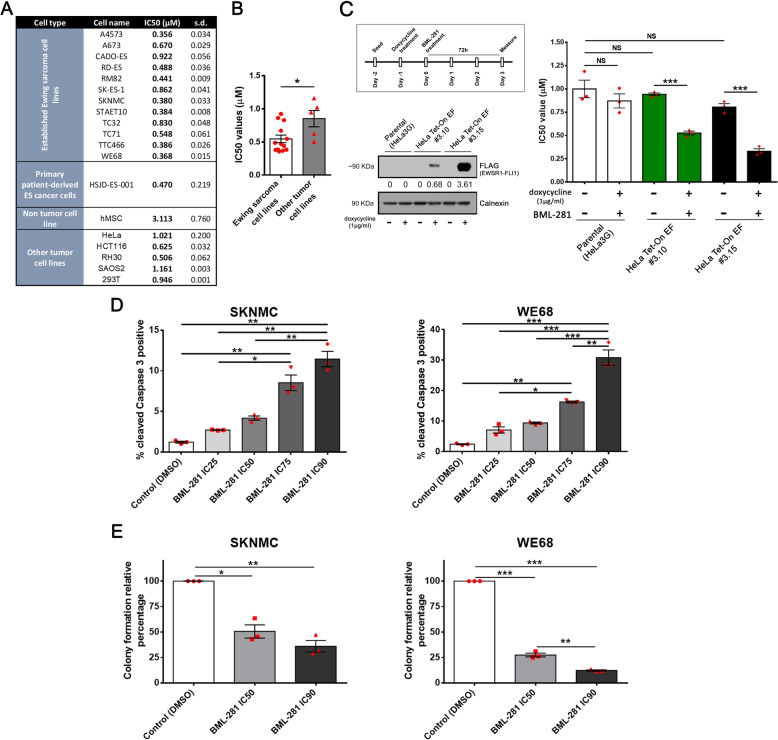
EWS cell lines showed a remarkably sensitivity to selective HDAC6 inhibition. **A** Cell viability assessment by 50% inhibitory concentration (IC50) of 13 EWS cell lines, one non-tumor cell line, and 5 non-EWS cancer cell lines, after 72 h exposure to BML-281. **B** Statistical comparison of the average sensitivity to IC50 HDAC6 inhibition between the two cell lines groups. **C** A schematic representation of the performed protocol for *EWSR1-FLI1* induction (upper left panel), and confirmation of the EWSR1-FLI1 ectopic expression protein by western blotting using HeLa cells treated with doxycycline (lower left panel). Numbers below blots represent densitometric quantification of bands, normalized to endogenous bands. Statistical comparison of sensitivity to HDAC6 inhibition with ectopic EWSR1-FLI1 protein expression and no expression in two clones with inducible chimeric protein-expression system in HeLa cells (right panel). **D** Cleaved-caspase 3 level assessment in SKNMC and WE68 cells treated with BML-281 in dose-dependent manner for 48 h and analyzed by flow cytometry. **E** Effects of HDAC6 inhibition on the anchorage-independent growth capacity of the EWS cell lines (SKNMC and WE68) by soft-agar colony formation assay. *P < 0.05; **P *<* 0.01; ***P < 0.001.

To investigate the key HDACs that control EWS sensitivity to HDACis, we performed a screening assay for 43 compounds that affect HDAC activity (#BML-2836, Enzo). We used seven EWS cell lines to estimate the proliferation inhibition capacity of these compounds (Supplementary Fig. 1A). The analysis revealed that only trichostatin A (a pan-HDACi) and BML-281 (a selective HDAC6 inhibitor) had an IC50 value below 1.5 μM for all seven EWS cell lines (Supplementary Fig. 1B). Due to the well-documented toxicity and side effects of pan-HDACis, we selected BML-281 as a potent promising HDACi for further analysis. We next expanded the list panel to thirteen EWS cell lines and validated the results of the compound screen for cells treated with BML-281. The median IC50 values in EWS cell lines (IC50 = 0.5465 µM) were significantly below those obtained for other tumor entity cell lines (IC50 = 0.8518 µM) and the non-tumor cell line hMSC (IC50 = 3.113 µM) (Fig. 1A, B).

We next analyzed whether EWS cell sensitivity to BML-281 correlated with the level of HDAC6 protein expression. We demonstrated that there were no statistical differences in the levels of HDAC6 protein expression in the EWS cell lines as compared to other tumor cell lines (Supplementary Fig. 1C, D). Therefore, our results showed that the sensitivity of EWS cells to selective inhibition of HDAC6 via BML-281 did not involve changes in its expression (Supplementary Fig. 1E). The significant sensitivity to HDAC6 inhibition in the EWS cell lines could be due to a role of the fusion protein as a regulator of the epigenetic landscape, as it is well known that EWSR1-FLI1 modulates HAT and HDAC activities and subsequently affects their targets [8]. To investigate whether the fusion protein expression “per se” sensitizes EWS cells to HDAC6 inhibition, we induced the chimeric protein expression in an ectopic model using HeLa cells. Notably, we previously demonstrated that EWSR1-FLI1 expression in HeLa cells mirrors its function in EWS [21]. The EWSR1-FLI1 expression in the two selected clones (#3.10 and #3.15) increased the sensitivity with respect to a non-induced system and reduced IC50-BML-281 values (Fig. 1C). In contrast, IC50 value in parental HeLa cells was not modified by doxycycline treatment. These results suggest that EWSR1-FLI1 expression in the doxycycline-independent HeLa model increased the sensitivity of HeLa cells to BML-281 treatment.

As BML-281 impairs proliferation in EWS cell lines, we analyzed its effects on apoptotic cell death and clonogenicity. We first determined the inhibition of HDAC6 effects on cleaved caspase 3 induction in EWS cells after a 48 h exposure to BML-281. We detected a significant induction of cleaved caspase 3 in different EWS cell lines, mainly at medium (IC50, IC75) and high (IC90) concentrations of BML-281 (Supplementary Fig. 1F), after a 48 h exposure as compared to the control (Fig. 1D). We then investigated the residual effects of HDAC6 inhibition in a clonogenic assay (which measures the ability to form 3D-colonies in a soft agar medium) after a 24 h exposure to medium or high doses of BML-281. At the IC50 and IC90 concentrations, colony formation was significantly reduced to 50.59% and 36.01%, respectively, in SKNMC cells, and to 27.42% and 12.22%, respectively, in WE68 cells, as compared to non-treated cells (Fig. 1E). Our results indicated that the selective inhibition of HDAC6 by BML-281 induced apoptosis and reduced the capacity of cells for tumorigenesis in vitro.

### Nuclear HDAC6 activity inhibits EWSR1-FLI1 expression in EWS cell lines

**Fig. 2:**
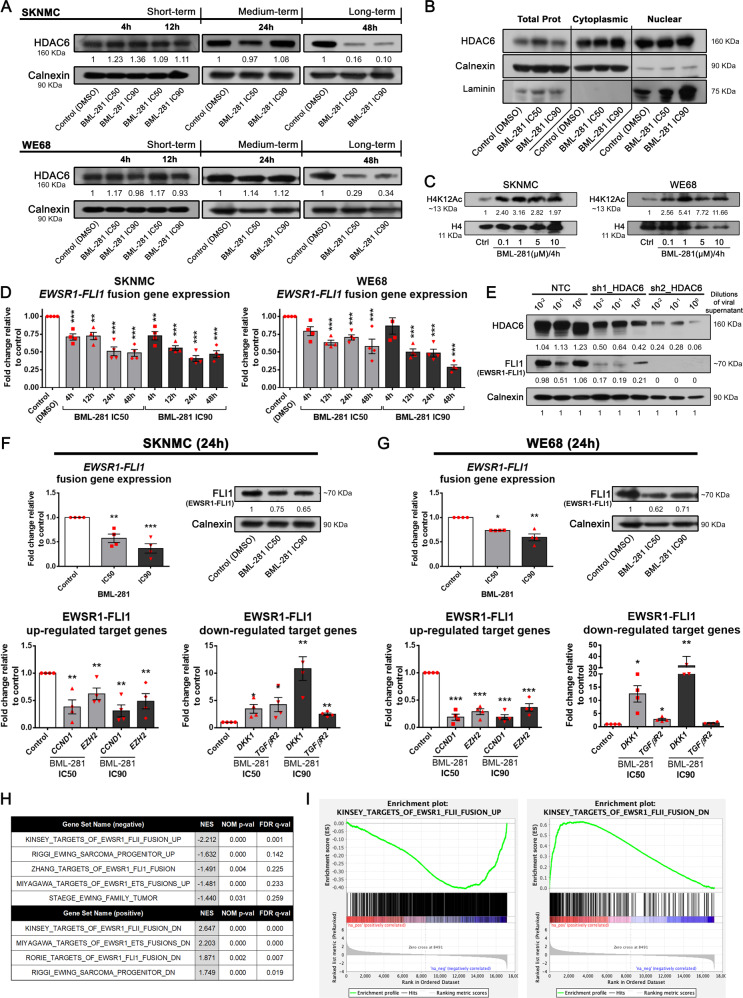
Inhibition of HDAC6 activity induces EWSR1-FLI1 downexpression and reduced its oncogenic activity. **A** Time course experiments (short-, medium-, and long-term) of HDAC6 protein expression was evaluated by immunoblotting using extracts from SKNMC and WE68 EWS cell lines treated with IC50 or IC90 concentrations of BML-281. **B** Subcellular fractionation and immunoblotting evaluation of HDAC6 localization in both cytoplasm and nucleus fractions. **C** Dose-dependent evaluation of H4K12 acetylation level in SKNMC and WE68 cell lines exposed to increasing concentrations of BML-281 (0.1, 1, 5, and 10 μM) for 4 h. **D** RT-qPCR analysis of *EWSR1-FLI1* mRNA level in SKNMC and WE68 EWS cell lines treated with BML-281 (IC50 and IC90) in time-course experiments. **E** Immunoblot assessment of HDAC6 and EWSR1-FLI1 protein expression levels after HDAC6 depletion by two shRNA constructs. **F**, **G** RT-qPCR and immunoblot assessment of mRNA and protein expression levels of EWSR1-FLI1 (**F**, **G**, upper panels), and EWSR1-FLI1 regulating target genes (**F**, **G**, lower panels) after 24 h of BML-281 treatment at IC50 and IC90 concentrations in the SKNMC or WE68 cell line, respectively. **H** GSEA C2_MSigDB analysis showed the overlap between genes that were significantly repressed or induced by BML-281 in EWS cell lines at EWSR1-FLI1 target genes signatures. **I** Enrichment plots with the best enrichment based on the |NES| (normalized enrichment score) are shown. Numbers below blots represent densitometric quantification of bands, normalized to endogenous bands and referred to their respective controls (DMSO band from the same time point). *P < 0.05; **P *<* 0.01; ***P < 0.001.

We then analyzed the role of HDAC6 by measuring its activity. We first evaluated HDAC6 expression in response to BML-281 at different time points. Early time points of BML-281 exposure did not affect protein or mRNA HDAC6 expression levels in SKNMC or WE68 cells, as shown by immunoblotting, RT-qPCR, and RNA-seq results (Fig. 2A, Supplementary Fig. 2A, and Supplementary Table 1). However, HDCA6 expression levels were reduced after a long-term (48 h) exposure to BML-281 (Fig. 2A). Although the inhibitor BML-281 did not decrease HDAC6 expression with short-term exposure, HDAC6 activity was impaired. In both EWS cell lines, α-tubulin acetylation was strongly induced after HDAC6 inhibition in EWS cells lines after short-term treatment and maintained over time (Supplementary Fig. 2B).

We confirmed the presence of both nuclear and cytoplasmic HDAC6 in EWS cell lines (Fig. 2B) with no changes in HDAC6 expression levels after a 24 h BML-281 treatment in either cytoplasm or nucleus. We also explored nuclear HDAC6 activity in EWS by evaluating the deacetylation of H4K12ac, which we have previously shown to be the specific histone-residue target [17]. HDAC6 inhibition strongly induced H4K12 residue acetylation in EWS cell lines (Fig. 2C). Further, we confirmed the nuclear HDAC6 role by using the enrichment_GO analysis (Supplementary Table 2). These bioinformatics analyses interestingly revealed that early HDAC6 inhibition (at 4 h) induced changes in expression of genes involved in histone and chromatin modifications and in regulation of DNA binding of transcription factors. In addition, we observed that nuclear genes enrichment was maintained beyond 24 h (Supplementary Table 2). These data support the nuclear role of HDAC6 in EWS cells.

Due to nuclear localization of HDAC6 and its role in EWS cell lines, we further evaluated if HDAC6’s nuclear activity affects the EWSR1-FLI1 protein. Our results showed significant downregulation of *EWSR1-FLI1* mRNA at early time points after BML-281 treatment (Fig. 2D). We also identified that BML-281 reduced the fusion protein expression level at 12 h in both EWS cell lines, and this reduction was maintained over time (Supplementary Fig. 2C). To verify the specificity of BML-281 in reducing the levels of EWSR1-FLI1, HDAC6 expression was depleted by two shRNA constructs (Fig. 2E). Indeed, targeted HDAC6 downregulation specifically reduced the aberrant transcription factor expression, confirming that HDAC6 positively regulated EWSR1-FLI1.

We next checked the downstream effects of HDAC6’s regulation of EWSR1-FLI1 on expression of known EWSR1-FLI1 target genes. In parallel, we evaluated the downregulation of EWSR1-FLI1 (at both mRNA and protein levels). Genes that are canonically activated by upregulated EWSR1-FLI1 (*CCND1* and *EZH2*) were downregulated by HDAC6 inhibition, whereas those which are classically repressed (*DKK1* and *TGFβR2*) were upregulated in both EWS cell lines after treatment (Fig. 2F, G, and Supplementary Fig. 2D, E).

To determine the relevance of EWSR1-FLI1 depletion by HDAC6 inhibition, we expanded our analysis to include genes that were significantly modulated by BML-281 in the SKNMC and WE68 cell lines (Supplementary Table 1). After conducting a pre-ranked study with GSEA software, we compared our list of differentially expressed genes (DEG) against the C2_MSigDB signature database. We observed a statistically significant enrichment in EWS signatures (Kinsey, Miyagawa, Riggi, Rorie or Zhang), with negative normalized enrichment scores (NES) for mostly upregulated genes (UP gene sets), and positive NES for downregulated genes (DN gene sets), in our treated EWS cell lines (Fig. 2H, I, and Supplementary Table 3). These data revealed an inverse response to de novo expression of EWSR1-FLI1 in a previously described ectopic model [21]. GSEA of DEG analyses confirmed that the gene signature induced by HDAC6 inhibition recapitulated the molecular features, and therefore the biology of the specific chimeric fusion depletion in EWS cell lines. Altogether, our results indicated that EWSR1-FLI1 is downregulated by HDAC6 inhibition.

### HDAC6 regulates *EWSR1-FLI1* expression through SP1/P300 binding to the *EWSR1* promoter

**Fig. 3:**
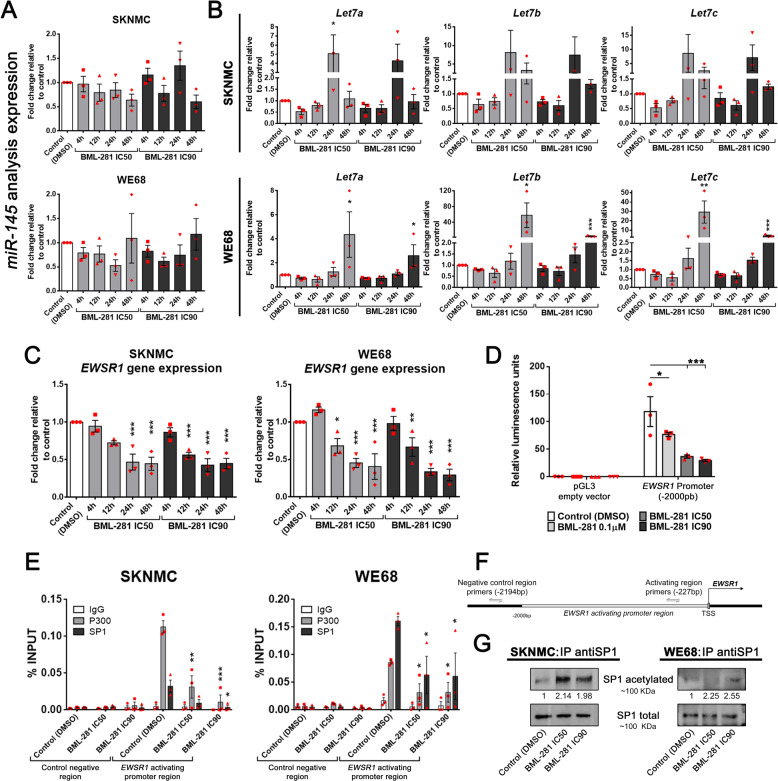
HDAC6 regulates *EWSR1-FLI1* and endogenous *EWSR1* expression through SP1/P300 binding to their promoters. **A** RT-qPCR analysis of *miR-145* levels in SKNMC and WE68 EWS cell lines treated with BML-281. **B** RT-qPCR analysis of the expression levels of *Let-7* family members in SKNMC and WE68 EWS cell lines treated with BML-281 treatment. **C** RT-qPCR analysis of *EWSR1* mRNA expression of the non-translocated gene in SKNMC and WE68 EWS cell lines treated with BML-281. **D** Evaluation of *EWSR1* promoter region-dependent transcriptional activation with luciferase reporter constructs containing the region of 2000 bp upstream of the *EWSR1* transcription start site (TSS), in a dose-dependent BML-281-treatment of SKNMC cells for 4 h. **E**, **F** ChIP and qPCR analysis of SP1 and/or P300 binding levels at *EWSR1* activating promoter region in SKNMC and WE68 EWS cell lines treated with BML-281. % Input indicates enrichment ratio of immunoprecipitated samples relative to input. The IgG antibody was used as a control for unspecific binding in ChIP assays. Binding sites of the primers used are shown schematically (**F**). **G** SP1 pull-down and evaluation of lysine acetylated levels against total SP1 protein expression in both SKNMC and WE68 cell lines treated for 24 h with increasing concentrations of BML-281 (IC50 and IC90). Numbers below blots represent densitometric quantification of bands, normalized to endogenous bands (total SP1) and referred to their respective controls (DMSO). *P < 0.05; **P *<* 0.01; ***P < 0.001.

Increased transcription levels of mirRNA-145 and Let-7 family members reduces the transcription levels of *EWSR1-FLI1* [22, 23]. Therefore, we explored whether *EWSR1-FLI1* regulation by HDAC6 inhibition is due to the transcription modulation of them. *miR-145* expression levels were not significantly affected by BML-281, regardless drug concentrations or exposure times (Fig. 3A). However, we observed an increase of *Let-7* genes expression at 24 h, followed by reduction at 48 h, in the SKNMC cell line; and only a significant upregulation of the *Let-7* genes family at 48 h in the WE68 cell line (Fig. 3B). These results suggested that Let-7 could contribute, at least in part, to *EWSR1-FLI1* downregulation induced by BML-281 in EWS cell lines only after long-term of HDAC6 inhibition. However, HDAC6 inhibition cannot explain *EWSR1-FLI1* inhibition after short-term exposure to HDAC6 inhibition.

To investigate short-term regulation, we used the ectopic EWSR1-FLI1 expression model in HeLa cells [21]. Surprisingly, BML-281 treatment induced fusion protein overexpression (Supplementary Fig. 3A). This response rules out posttranscriptional regulation as a principal *EWSR1-FLI1* inhibition mechanism by BML-281 and suggests that the gene fusion promoter is a potential candidate. Based on this indirect evidence, we further evaluated the role of the *EWSR1* promoter as a principal element for HDAC6-mediated *EWSR1-FLI1* inhibition. Consistently, we analyzed the endogenous (non-translocated) *EWSR1* expression using specific previously reported primers [10]. We observed a statistically significant downregulation after 12 h or 24 h of BML-281 treatment in WE68 and SKNMC cells, respectively (Fig. 3C); this is in agreement with the inhibition of *EWSR1-FLI1* expression shown by both RT-qPCR and RNA-seq (Fig. 2D and Supplementary Table 1). To confirm *EWSR1*/*EWSR1-FLI1* promoter regulation by HDAC6, we designed a luciferase-reporter construct whose promoter comprised of 2000bp upstream of the *EWSR1* transcription start site. We found a significant dose-dependent inhibition of luciferase expression (Fig. 3D). Altogether, our results demonstrated that regulation of expression of both *EWSR1* and *EWSR1-FLI1* by HDAC6 inhibition was mediated through the promoter.

To better understand how HDAC6 regulates the *EWSR1*/fusion gene promoter, we conducted an in silico prediction of possible intermediate elements that could participate in this regulation. We found that the p300 protein is situated between HDAC6 and EWSR1 (Supplementary Fig. 3B). Previous studies demonstrated that EWSR1-FLI1 recruits p300 for activating gene expression [6]. We therefore analyzed the physical interactions between HDAC6 and the fusion protein to exclude a possible positive feedback loop. Our results showed that there was no direct protein–protein binding (Supplementary Fig. 3C), suggesting the existence of protein(s) that facilitate p300 binding to the fusion protein promoter. Notably, it has been demonstrated that SP1 can recruit p300 to activate gene transcription [24–26]. Further, SP1 is a *EWSR1-FLI1* positive regulator [27]. In addition, the in silico analysis demonstrated that HDAC6, EWSR1, and SP1 are interconnected through p300 (Supplementary Fig. 3D). First, we evaluated *SP1* expression by both RNA-seq and RT-qPCR. Our results showed a slight downregulation of *SP1* after BML-281 treatment (Supplementary Table 1 and Supplementary Fig. 3E) that was however not enough to explain the inhibition of fusion gene expression. To test whether HDAC6 regulates SP1 binding to *EWSR1* and *EWSR1-FLI1* promoters independently of the modulation of SP1 expression level, we analyzed binding of SP1 as well as of its coactivator p300 at activating region of *EWSR1* promoter (–2000 bp – 0; *EWSR1* transcription start site [28]) by chromatin immunoprecipitation. We showed that SP1 and p300 binding was impaired by HDAC6 inhibition in both EWS cell lines analyzed (Fig. 3E, F). Lysine acetylation in SP1 is associated with its reduced DNA-binding capacity [29]. Therefore, we analyzed the alteration of lysine acetylation status of SP1 under BML-281 treatment through a pull-down assay. We demonstrated increased total lysine acetylation of SP1 by HDAC6 inhibition in both EWS cell lines (Fig. 3G). Together, these results support that HDAC6 regulates, at least partially, the expression of *EWSR1*/*EWSR1-FLI1* through SP1-P300 complex binding to its promoters.

### High HDAC6 expression in EWS tumor samples was associated with poor prognosis

**Fig. 4:**
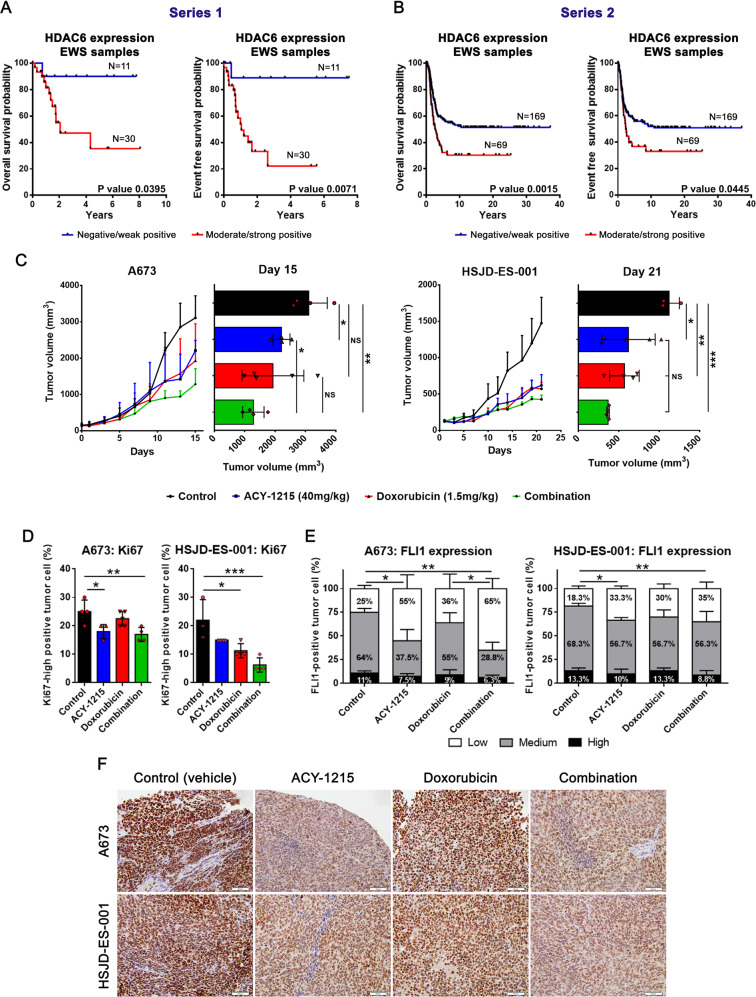
HDAC6 overexpression correlates with poor prognosis in EWS patients, and in vivo combined treatment reduces EWS tumor growth. **A**, **B** HDAC6 Kaplan–Meier plot with OS and DFS, according to the transcript. IHC expression in primary EWS tumor samples showed statistically significant differences between low- and high-HDAC6-expressing tumors in two independent series (series 1 and 2). **C** Tumor growth was monitored in A673 and HSJD-ES-001 xenografts models after single-agent or combination therapies. Tumor volumes (mm^3^) were measured after 15 days (A673) or 21 days (HSJD-ES-001) of treatment. **D** Quantification of Ki67-high positively-labeled nuclei percentage after 15 or 21 days of treatment. **E** Anatomopathological quantification of FLI1 expression after 15 or 21 days of treatment, with three levels of expression threshold set: low, medium, and high. **F** Immunohistochemical staining of FLI1 in xenograft tumor samples treated with ACY-1215 and doxorubicin, alone or in combination (40× magnifications). *P < 0.05; **P *<* 0.01; ***P < 0.001.

We evaluated the prognostic impact of HDAC6 expression levels in EWS tumor samples by immunohistochemistry (IHC). Specific HDAC6 staining was found in both the nucleus and cytoplasm of EWS tumor cells (Supplementary Fig. 4A). Due to the heterogeneous expression of HDAC6, Kaplan–Meier analysis was conducted, grouping patient samples with negative/weak intensity versus moderate/strong intensity. EWS patients with moderate/strong intensity of HDAC6 had worse overall survival and disease-free survival rates as compared to the negative/weak intensity group (Fig. 4A). To validate the findings obtained in our local series (series 1), we accessed a large cohort of EWS samples recruited in the multicenter Prothets and EuroBoNet projects (series 2), confirming that HDAC6 overexpression correlates with a poor prognosis in EWS patients (Fig. 4B).

### HDAC6 inhibition impairs EWS tumor growth in vivo

Given the prognostic significance of HDAC6 overexpression in EWS patients, we hypothesized that its inhibition could be a promising alternative treatment. Therefore, we first conducted an in vitro experiment to evaluate the effect of the combination of doxorubicin (a cytotoxic drug used in standard EWS chemotherapy treatment) and HDAC6 inhibition (using BML-281). A strongly synergistic effect (0.1 < combination index (CI) ≤ 0.7) was observed in the proliferation inhibition in both established and primary EWS cell line cultures from EWS patient-derived xenografts (PDX) (Supplementary Fig. 4B).

Next, we explored the therapeutic potential of HDAC6 inhibition (alone or in combination) in vivo. Mice were divided randomly into four groups: control (vehicle), ACY-1215 (50 mg/kg), doxorubicin (1.5 mg/kg), or both ACY-1215 and doxorubicin (Supplementary Fig. 4C). ACY-1215 (also known as ricolinostat) is a potent and selective HDAC6 inhibitor [13, 30]; we used it in the in vivo experiment based on its antitumor effects and efficacy, both alone and in combination with various conventional treatments for cancer [31–33] and in several clinical trials [13, 30]. Treatment was well tolerated in mice, with no weight loss (Supplementary Fig. 4D), or overall tissue alteration by histopathology (Supplementary Fig. 4E). After three weeks of therapy tumor volume was significantly reduced to similar degrees in both cell lines for the monotherapy treatment groups (ACY-1215 or doxorubicin) as compared to the control group. Notably, the combined treatment induced a deeper reduction in tumor size than either monotherapy alone (Fig. 4C).

Histopathological examination of tumors reveled no morphological alterations of EWS cells in any analyzed samples (Supplementary Fig. 4F). However, the percentage of high Ki67-positive labeled cell populations in the combination treatment group was significantly lower than that of the control group, and both monotherapy groups had levels between the two (Fig. 4D). Finally, we evaluated if the in vivo assay replicated the EWSR1-FLI1 depletion induced by HDAC6 inhibition in vitro. We observed that ACY-1215 treatment reduced EWSR1-FLI1 expression levels in tumor samples, both as a monotherapy or in combination with doxorubicin (Fig. 4E, F). While the reduction of EWSR1-FLI1 expression was higher in the combination therapy than in the ACY-1215 monotherapy, this difference was not statistically significant (Fig. 4E).

Collectively, these findings indicated that HDAC6 inhibition delays tumor growth in vivo and suggest that anti-tumor activity was mediated, at least in part, through its inhibition of *EWSR1-FLI1* oncogene expression.

## Discussion

Inhibiting the oncogenic fusion protein and restoring epigenetic changes could be a promising therapeutic alternative for EWS patients [2, 3, 34, 35]. Due to pan-HDACis effects on nonspecific targets [11], we hypothesized that it would be more appropriate to evaluate the effects of selective HDACis, which could maintain the effectiveness on EWSR1-FLI1 depletion while simultaneously reducing treatment toxicity [9, 11, 36, 37]. We thus performed a screen of 43 epigenetic drugs, most of them known to modulate HDAC activity. Our results revealed that EWS cell lines were remarkably sensitive to BML-281, a specific HDAC6 inhibitor. In fact, EWS cell lines were more sensitive to HDAC6 inhibition than other malignant cell lines and the non-tumor cell line hMSC (which are defined as the putative cells of origin for EWS [38]). This emphasizes the importance of HDAC6 in EWS malignancy. Further, we revealed that the sensitivity of EWS cells to HDAC6 inhibition was associated with the presence of EWSR1-FLI1 in the ectopic HeLa model [21] after BML-281 treatment, but not with the levels of HDAC6 expression. Thus, the acquisition of this great sensitivity could be explained by the chimeric protein redrawing the epigenetic landscape, and thereby modulating the specific binding sites of HDACs [39]. The specific HDAC targets regulated by EWSR1-FLI1 activity provide advantages to EWS tumor cells, and blockade of this regulation via HDAC inhibition could specifically sensitize EWS cells to BML-281 treatment.

We demonstrated the nuclear presence of HDAC6 in EWS cell lines by cell fractionation, and subsequently its nuclear activity (H4K12 acetylation modulation). Given that nuclear activity of HDAC6 and EWS sensitivity to HDAC6 inhibition were associated with the presence of EWSR1-FLI1, we explored a possible role of HDAC6 in the regulation of EWSR1-FLI1 expression. We observed that BML-281 treatment significantly downregulated *EWSR1-FLI1* mRNA level after treatment. In line with EWSR1-FLI1 downregulation, fusion-induced and fusion-repressed targets were down- and upregulated, respectively. Additionally, the expression signature induced by BML-281 in EWS cell lines as compared to the GSEA gene sets (C2_MSigDB) revealed a significant correlation with several previously published EWSR1-FLI1 target gene signatures. This response would explain why using pan-HDACis, such as SAHA or FK228, inhibits the driver of EWS [8, 10], due to the inhibition of HDAC6 activity by these drugs, as previously described [40, 41].

The next step was to understand how HDAC6 regulates EWSR1-FLI1 expression. *Let-7* genes modulation could likely contribute to *EWSR1-FLI1* regulation as a late effect of HDAC6 inhibition. However, another mechanism would be the main regulator of chimeric protein under the action of HDAC6 inhibition, both after short- and long-term treatment times, as previously observed. Indeed, we showed that the endogenous (non-translocated) *EWSR1* gene was also downregulated by HDAC6 inhibition, similar to inhibition of the fusion gene. Conversely, BML-281 treatment induced overexpression of the fusion protein in HeLa model. This excludes post-transcriptional regulation of fusion gene mRNA being a mechanism of *EWSR1-FLI1* depletion via HDAC6 inhibition; rather, it points to *EWSR1* promoter as a possible HDAC6 target (as it is the differentiating element in this ectopic model with respect to the endogenous *EWSR1-FLI1* gene in EWS cell lines). Overall, these results suggest that regulation of the fusion gene by HDAC6 occurred via the *EWSR1* promoter (the homologous sequence in both endogenous and fusion genes in EWS). We verified this relationship using a gene reporter assay showing a dose-dependent luciferase gene inhibition (under the *EWSR1* promoter) by BML-281 treatment.

**Fig. 5:**
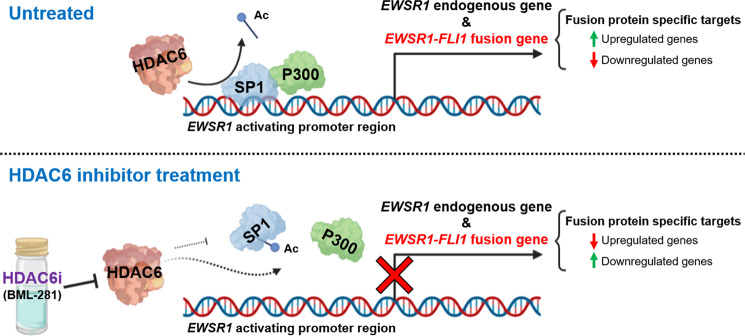
Schematic representation of regulation of *EWSR1* and *EWSR1-FLI1* transcription by SP1 and p300 binding complexes in both homologous promoter regions. HDAC6 modulates acetylation of SP1 as well as its binding to the *EWSR1*/*EWSR1-FLI1* activating promoter regions. In turn, HDAC6 increases SP1/p300 complex binding by deacetylating SP1. HDAC6 inhibition allows dissociation of the SP1/p300 complex by increased acetylation of SP1, with a subsequent decrease in both the expression of EWS fusion gene transcription and its protein activity, leading to modulation of a specific EWS target genes. Figure created with www.BioRender.com.

As HDAC inhibition induces transcription activation by restoring the acetylation levels of histones and non-histone proteins [9], an intermediate element between HDAC6 activity and *EWSR1* promoter inhibition is necessary. Recent studies have demonstrated that SP1, a relevant transcription factor in cancer [42], regulates *EWSR1-FLI1* [27]. Furthermore, Giogi et al. showed that SP1 depletion induces downregulation of the fusion gene and suggested that it is regulated via the fusion gene promoter [27]. Here, we demonstrated that HDAC6 modulates binding of SP1 to the *EWSR1*/*EWSR1-FLI1* promoters. As a transcription factor, SP1 is able to recruit other elements, such as p300, to activate gene transcription [24–26]. Additionally, p300 binds to the *EWSR1* promoter in the absence of HDAC6 inhibitor (inducing *EWSR1*/fusion gene transactivation). Inhibition of HDAC6 releases both factors, downregulating both *EWSR1-FLI1* and the endogenous *EWSR1* expression. Loss of SP1 affinity to the *EWSR1* promoter by HDAC6 inhibition might be due to the acetylation of the only known residue, lysine 703, which resides in its DNA-binding domain [29]. Waby et al. demonstrated that SP1 acetylation at lysine-703 releases this transcription factor from specific promoter targets [29]. Indeed, we observed increased SP1 lysine acetylation after BML-281 treatment. We suggest that HDAC6 inhibition induces acetylation of SP1 DNA-binding domain. This induction would then promote the release of SP1 and the recruited p300 from the *EWSR1*/*EWSR1-FLI1* promoters. Our results show for the first time a specific epigenetic mechanism that regulates *EWSR1-FLI1* expression through its promoter, with HDAC6 playing a central role, and confirm the importance of using a selective HDAC6 inhibitor in EWS (Fig. 5). This is in accordance with recent epigenetic marks that have been described to activate the fusion gene transcription: enrichment of H3K4me3, H3K9ac, and H3K27ac, which can result from SP1/P300 activity at the fusion gene promoter [43]. Whether histone modification H4K12ac, a specific HDAC6 target, plays a role in the *EWSR1-FLI1* regulation remains to be investigated.

We demonstrated that HDAC6 overexpression correlated with poor outcome in two independent series of EWS patients. Accordingly, we suggest the possible use of HDAC6 inhibition as an alternative treatment in EWS clinical trials. Using ACY-1215 treatment (a selective HDAC6 inhibitor that has successfully passed a phase-Ib clinical trial for refractory multiple myeloma [44]), we observed a significant tumor growth inhibition in xenograft models. HDAC6 inhibitors are known to affect a myriad of cellular functions essential in tumor progression [13]. Alternatively, their effect in EWS could also be explained by the fusion protein modulation via inhibition of HDAC6 activity, leading to alteration of HDAC aberrant chromatin accessibility regulated by the fusion protein in EWS [39]. Thus, HDAC6 inhibition could be used as a selective therapy against the EWSR1-FLI1 epigenetic signature [36].

To increase its efficiency HDAC6 selective inhibitors have been combined with DNA damaging or immunomodulatory drugs in several malignancies [45–47]. Here, we combined ACY-1215 with doxorubicin, a standard drug for EWS patient treatment. This combination enhanced tumor growth inhibition as compared to either monotherapy. Moreover, HDAC6 inhibition in vivo by ACY-1215 treatment downregulated EWSR1-FLI1 protein, both in monotherapy and in combination, without animal toxicity. Indeed, HDAC6-deficient mice are viable and fertile [20]. We therefore believe that HDAC6 depletion can be a safe strategy for EWS patient treatment.

We conclude that selective HDAC6 inhibition, which inhibits the oncogenic EWSR1-FLI1 fusion protein and takes aberrant epigenetic changes back, could be a promising, selective, and safe EWS therapeutic alternative.

## Materials and methods

### Epigenetic drug library screening

A4573, CADO-ES, RDES, SK-ES-1, SKNMC, STAET 2.1, and TTC466 EWS cells were treated with 43 different epigenetic drugs (#BML-2836, Enzo). Proliferation was measured at 72 h after treatment by MTT assay. This screening was conducted by the Mejoran Lab (Madrid, Spain).

### Acid histone extraction

Cells were washed with ice-cold 5 mM sodium butyrate-PBS (1×). Centrifuged and resuspend cells in hypotonic extraction buffer (10 mM Tris-Cl pH8.0, 1 mM KCl, 1.5 mM MgCl_2_, and 1 mM DTT) were supplemented with protease inhibitor. Cells were lysed on ice for 30 min and centrifuged for 10 min at 10,000 × *g* for nucleus precipitation. Supernatants were discarded and eluted in 0.4 M H_2_SO_4_, incubated for 30 min on ice, and then centrifuged 10 min at 16,000 × *g*. Supernatants were treated with 33% trichloroacetic acid (v/v) and incubated overnight at –20°C to precipitate histones. Pellets containing histones were washed twice with cold acetone and then centrifuged 5 min at 16,000 × *g*, and supernatants were removed and air dried. Histone pellets were resuspended in ddH_2_O.

### Short hairpin RNA (shRNA) HDAC6

Two pLKO.1-shRNA constructions against the HDAC6 transcript were selected from the MISSION shRNA collection (SIGMA Aldrich). The constructs references were TRCN0000314976 for sh1_HDAC6 and TRCN0000004839 for sh2_HDAC6. An empty MISSION pLKO.1-puro (SIGMA Aldrich) was included as a control of the off-target effects. Transfection of 293 T cells and subsequent transduction were carried out using the protocol described previously [48].

### Luciferase reporter assay

The pGL3-Basic vector (Promega) was used, and the *EWSR1*-activating promoter region (–2000 bp to TSS [28]) was successfully cloned upstream of the luciferase gene. pGL3-Empty vector was included as a control for off-target effects. Cells were seeded in 96-well plates at 24 h before transfection. Both constructs were transfected with Lipofectamine2000 (Invitrogen) together with pRL-TK Renilla (100 ng/well) to normalize for transfection efficiency. Cells were treated for 4 h with different doses of BML-281 or control medium (DMSO 0.1%) and then collected at 4 h after treatment. Cell lysates were assessed using the Dual-Luciferase Reporter Assay System (#E1910; Promega, Madison, WI, USA).

### Clinical samples and tissue microarrays (TMAs)

Series 1 EWS samples had been obtained between 1991 and 2017 and comprised 93 paraffin-embedded tumor samples from 52 patients. Patient characteristics are summarized in Supplementary Table 4. Approval of the Ethics Committee of our institution was obtained before including samples and data into the HUVR-IBiS Biobank. Series 2 EWS samples consists of a retrospective cohort of EWS collected in the framework of the Prothets and EuroBoNet projects [49, 50]. Clinicopathologic characteristics of this specific cohort of patients (*n* = 341) are summarized in Supplementary Table 5. Representative malignant areas from samples were carefully selected from the stained sections of each tumor, and two 1 mm diameter tissue cores were obtained from each sample.

### In vivo mouse xenograft models

Suspensions A673 and HSJD-ES-001 containing 4.5 × 10^6^ living cells were in a 0.2 ml final volume composed of medium and Matrigel in a 1:1 proportion; these were injected subcutaneously in five-week-old CB17-SCID female mice (Envigo). Mice were randomized into four groups, and treatments started when the mean tumor volume reached 200 mm^3^. Mice were injected intraperitoneal in accordance with the previously cited protocol (Supplementary Fig. 4C). Tumor sizes were monitored, and mice were sacrificed when tumor volumes reached tolerable size limits or at 21 days of treatment. In vivo studies were performed with approval of the Institutional Animal Research Ethics Committee, in conformation with the Spanish Royal Decree 1386/2018.

Additional methods are described in Supplementary Material and Methods. Materials and reagents used in the present study are described in Supplementary Table 6.

## Supplementary information

Supplemental Data

Supplementary Table 1

Supplementary Table 2

Supplementary Table 3

Supplementary Table 4

Supplementary Table 5

Supplementary Table 6

Supplemental Materials and Methods

## Data Availability

Our RNA-seq data have been deposited in the NCBI Sequence Read Archive (SRA) as PRJNA673347.

## References

[1] Grünewald TGP, Cidre-Aranaz F, Surdez D, Tomazou EM, de Álava E, Kovar H (2018). Ewing sarcoma. Nat Rev Dis Prim.

[2] Herrero-Martín D, Osuna D, Ordóñez JL, Sevillano V, Martins AS, Mackintosh C (2009). Stable interference of EWS-FLI1 in an Ewing sarcoma cell line impairs IGF-1/IGF-1R signalling and reveals TOPK as a new target. Br J Cancer.

[3] Martinez-Lage M, Torres-Ruiz R, Puig-Serra P, Moreno-Gaona P, Martin MC, Moya FJ (2020). In vivo CRISPR/Cas9 targeting of fusion oncogenes for selective elimination of cancer cells. Nat Commun.

[4] Prieur A, Tirode F, Cohen P, Delattre O (2004). EWS/FLI-1 silencing and gene profiling of Ewing cells reveal downstream oncogenic pathways and a crucial role for repression of insulin-like growth factor binding protein 3. Mol Cell Biol.

[5] Sankar S, Bell R, Stephens B, Zhuo R, Sharma S, Bearss DJ (2013). Mechanism and relevance of EWS/FLI-mediated transcriptional repression in Ewing sarcoma. Oncogene.

[6] Riggi N, Knoechel B, Gillespie SM, Rheinbay E, Boulay G, Suvà ML (2014). EWS-FLI1 utilizes divergent chromatin remodeling mechanisms to directly activate or repress enhancer elements in Ewing sarcoma. Cancer Cell.

[7] Sanchez-Molina S, Figuerola-Bou E, Blanco ME, Sánchez-Jiménez M, Táboas P, Gomez-Gonzalez S (2020). RING1B recruits EWSR1-FLI1 and cooperates in the remodeling of chromatin necessary for Ewing sarcoma tumorigenesis. Sci Adv.

[8] Sakimura R, Tanaka K, Nakatani F, Matsunobu T, Li X, Hanada M (2005). Antitumor effects of histone deacetylase inhibitor on Ewing’s family tumors. Int J Cancer.

[9] Sun Y, Sun Y, Yue S, Wang Y, Lu F (2018). Histone deacetylase inhibitors in cancer therapy. Curr Top Med Chem.

[10] García-Domínguez DJ, Hontecillas-Prieto L, Rodríguez-Núñez P, Pascual-Pasto G, Vila-Ubach M, García-Mejías R (2018). The combination of epigenetic drugs SAHA and HCI-2509 synergistically inhibits EWS-FLI1 and tumor growth in Ewing sarcoma. Oncotarget.

[11] Hontecillas-Prieto L, Flores-Campos R, Silver A, de Alava E, Hajji N, Garcia-Dominguez DJ (2020). Synergistic enhancement of cancer therapy using HDAC inhibitors: opportunity for clinical trials. Front Genet.

[12] Lee YS, Lim KH, Guo X, Kawaguchi Y, Gao Y, Barrientos T (2008). The cytoplasmic deacetylase HDAC6 is required for efficient oncogenic tumorigenesis. Cancer Res.

[13] Seidel C, Schnekenburger M, Dicato M, Diederich M (2015). Histone deacetylase 6 in health and disease. Epigenomics.

[14] Liu Y, Peng L, Seto E, Huang S, Qiu Y (2012). Modulation of histone deacetylase 6 (HDAC6) nuclear import and tubulin deacetylase activity through acetylation. J Biol Chem.

[15] Wang Z, Zang C, Cui K, Schones DE, Barski A, Peng W (2009). Genome-wide mapping of HATs and HDACs reveals distinct functions in active and inactive genes. Cell.

[16] Yang CJ, Liu YP, Dai HY, Shiue YL, Tsai CJ, Huang MS (2015). Nuclear HDAC6 inhibits invasion by suppressing NF-kappaB/MMP2 and is inversely correlated with metastasis of non-small cell lung cancer. Oncotarget.

[17] García-Domínguez DJH-P L, Kaliszczak M, He M, Burguillos MA, Bekay R, Abdul-Salam VB et al. Novel nuclear role of HDAC6 in prognosis and therapeutic target for colorectal cancer. bioRxiv. 2020. https://www.biorxiv.org/content/10.1101/2020.11.02.356121v1.

[18] Kanno K, Kanno S, Nitta H, Uesugi N, Sugai T, Masuda T (2012). Overexpression of histone deacetylase 6 contributes to accelerated migration and invasion activity of hepatocellular carcinoma cells. Oncol Rep.

[19] Wang Z, Hu P, Tang F, Lian H, Chen X, Zhang Y (2016). HDAC6 promotes cell proliferation and confers resistance to temozolomide in glioblastoma. Cancer Lett.

[20] Zhang Y, Kwon S, Yamaguchi T, Cubizolles F, Rousseaux S, Kneissel M (2008). Mice lacking histone deacetylase 6 have hyperacetylated tubulin but are viable and develop normally. Mol Cell Biol.

[21] García-Domínguez DJ, Hontecillas-Prieto L, León EA, Sánchez-Molina S, Rodríguez-Núñez P, Morón FJ (2020). An inducible ectopic expression system of EWSR1-FLI1 as a tool for understanding Ewing sarcoma oncogenesis. PLoS One.

[22] Ban J, Jug G, Mestdagh P, Schwentner R, Kauer M, Aryee DN (2011). Hsa-mir-145 is the top EWS-FLI1-repressed microRNA involved in a positive feedback loop in Ewing’s sarcoma. Oncogene.

[23] Keskin T, Bakaric A, Waszyk P, Boulay G, Torsello M, Cornaz-Buros S (2020). LIN28B underlies the pathogenesis of a subclass of ewing sarcoma LIN28B control of EWS-FLI1 stability. Cell Rep.

[24] Azahri NS, Di Bartolo BA, Khachigian LM, Kavurma MM (2012). Sp1, acetylated histone-3 and p300 regulate TRAIL transcription: mechanisms of PDGF-BB-mediated VSMC proliferation and migration. J Cell Biochem.

[25] Formisano L, Guida N, Valsecchi V, Cantile M, Cuomo O, Vinciguerra A (2015). Sp3/REST/HDAC1/HDAC2 complex represses and Sp1/HIF-1/p300 complex activates ncx1 gene transcription, in brain ischemia and in ischemic brain preconditioning, by epigenetic mechanism. J Neurosci.

[26] Hung JJ, Wang YT, Chang WC (2006). Sp1 deacetylation induced by phorbol ester recruits p300 to activate 12(S)-lipoxygenase gene transcription. Mol Cell Biol.

[27] Giorgi C, Boro A, Rechfeld F, Lopez-Garcia LA, Gierisch ME, Schäfer BW (2015). PI3K/AKT signaling modulates transcriptional expression of EWS/FLI1 through specificity protein 1. Oncotarget.

[28] Moller E, Mandahl N, Iliszko M, Mertens F, Panagopoulos I (2009). Bidirectionality and transcriptional activity of the EWSR1 promoter region. Oncol Rep.

[29] Waby JS, Chirakkal H, Yu C, Griffiths GJ, Benson RS, Bingle CD (2010). Sp1 acetylation is associated with loss of DNA binding at promoters associated with cell cycle arrest and cell death in a colon cell line. Mol Cancer.

[30] Wang XX, Wan RZ, Liu ZP (2018). Recent advances in the discovery of potent and selective HDAC6 inhibitors. Eur J Med Chem.

[31] Amengual JE, Johannet P, Lombardo M, Zullo K, Hoehn D, Bhagat G (2015). Dual targeting of protein degradation pathways with the selective HDAC6 inhibitor ACY-1215 and bortezomib is synergistic in lymphoma. Clin Cancer Res.

[32] Cao J, Lv W, Wang L, Xu J, Yuan P, Huang S (2018). Ricolinostat (ACY-1215) suppresses proliferation and promotes apoptosis in esophageal squamous cell carcinoma via miR-30d/PI3K/AKT/mTOR and ERK pathways. Cell Death Dis.

[33] Santo L, Hideshima T, Kung AL, Tseng JC, Tamang D, Yang M (2012). Preclinical activity, pharmacodynamic, and pharmacokinetic properties of a selective HDAC6 inhibitor, ACY-1215, in combination with bortezomib in multiple myeloma. Blood.

[34] Nacev BA, Jones KB, Intlekofer AM, Yu JSE, Allis CD, Tap WD (2020). The epigenomics of sarcoma. Nat Rev Cancer.

[35] Scotlandi K, Hattinger CM, Pellegrini E, Gambarotti M, Serra M. Genomics and therapeutic vulnerabilities of primary bone tumors. Cells. 2020;9:968. 10.3390/cells9040968 PMC7227002 32295254

[36] de Nigris F, Ruosi C, Napoli C (2020). Clinical efficiency of epigenetic drugs therapy in bone malignancies. Bone.

[37] Tang F, Choy E, Tu C, Hornicek F, Duan Z (2017). Therapeutic applications of histone deacetylase inhibitors in sarcoma. Cancer Treat Rev.

[38] Tirode F, Laud-Duval K, Prieur A, Delorme B, Charbord P, Delattre O (2007). Mesenchymal stem cell features of Ewing tumors. Cancer Cell.

[39] Pattenden SG, Simon JM, Wali A, Jayakody CN, Troutman J, McFadden AW (2016). High-throughput small molecule screen identifies inhibitors of aberrant chromatin accessibility. Proc Natl Acad Sci USA.

[40] Furumai R, Matsuyama A, Kobashi N, Lee KH, Nishiyama M, Nakajima H (2002). FK228 (depsipeptide) as a natural prodrug that inhibits class I histone deacetylases. Cancer Res.

[41] Negmeldin AT, Padige G, Bieliauskas AV, Pflum MK (2017). Structural requirements of HDAC inhibitors: SAHA analogues modified at the C2 position display HDAC6/8 selectivity. ACS Med Chem Lett.

[42] Beishline K, Azizkhan-Clifford J (2015). Sp1 and the ‘hallmarks of cancer’. FEBS J.

[43] Montoya C, Rey L, Rodríguez J, Fernández MJ, Troncoso D, Cañas A (2020). Epigenetic control of the EWSFLI1 promoter in Ewing’s sarcoma. Oncol Rep.

[44] Yee AJ, Bensinger WI, Supko JG, Voorhees PM, Berdeja JG, Richardson PG (2016). Ricolinostat plus lenalidomide, and dexamethasone in relapsed or refractory multiple myeloma: a multicentre phase 1b trial. Lancet Oncol.

[45] Huang P, Almeciga-Pinto I, Jarpe M, van Duzer JH, Mazitschek R, Yang M (2017). Selective HDAC inhibition by ACY-241 enhances the activity of paclitaxel in solid tumor models. Oncotarget.

[46] Lee DH, Won HR, Ryu HW, Han JM, Kwon SH (2018). The HDAC6 inhibitor ACY1215 enhances the anticancer activity of oxaliplatin in colorectal cancer cells. Int J Oncol.

[47] Ray A, Das DS, Song Y, Hideshima T, Tai YT, Chauhan D (2018). Combination of a novel HDAC6 inhibitor ACY-241 and anti-PD-L1 antibody enhances anti-tumor immunity and cytotoxicity in multiple myeloma. Leukemia.

[48] Mackintosh C, Ordonez JL, Garcia-Dominguez DJ, Sevillano V, Llombart-Bosch A, Szuhai K (2011). 1q gain and CDT2 overexpression underlie an aggressive and highly proliferative form of Ewing sarcoma. Oncogene.

[49] Llombart-Bosch A, Machado I, Navarro S, Bertoni F, Bacchini P, Alberghini M (2009). Histological heterogeneity of Ewing’s sarcoma/PNET: an immunohistochemical analysis of 415 genetically confirmed cases with clinical support. Virchows Arch.

[50] Machado I, Lopez-Guerrero JA, Scotlandi K, Picci P, Llombart-Bosch A (2018). Immunohistochemical analysis and prognostic significance of PD-L1, PD-1, and CD8+ tumor-infiltrating lymphocytes in Ewing’s sarcoma family of tumors (ESFT). Virchows Arch.

