# Supplementary material for: Selective inhibition of HDAC6 regulates expression of the oncogenic driver EWSR1-FLI1 through the EWSR1 promoter in Ewing sarcoma
Source: Oncogene. 2021 Aug 3;40(39):5843–53. doi: 10.1038/s41388-021-01974-4 (PMC8484017; doi:10.1038/s41388-021-01974-4)
Supplement: Supplementary file 1 — Supplemental Data [file 41388_2021_1974_MOESM1_ESM.pdf]

SUPPLEMENTARY FIGURES AND LEGENDS

Supplementary Fig.1

A

No.	Cat. No.	CAS #	Compound Name	M.W.	Solvent	A4573 IC50 (Average)	CADO-ES IC50 (Average)	RD-ES IC50 (Average)	STAET 2.1 IC50 (Average)	SKNMC IC50 (Average)	TTC466 IC50 (Average)	SK-ES-1 IC50 (Average)
1	GR-309	58880-19-6	**Trichostatin A**	302.4	DMSO	0.0423	0.1518	0.0694	0.116	0.048	1.4396	0.3159
2	A-280	499-80-9	**2,4-Pyridinedicarboxylic Acid**	167.1	DMSO	>100	>100	>100	>100	>100	>100	>100
3	GR-343	78824-30-3	**Garcinol**	602.8	DMSO	6.9051	10.371	6.5598	20.4114	7.3219	18.449	21.9499
4	GR-331	5690-03-9	**Splitomicin**	198.2	DMSO	>100	>100	>100	>100	>100	>100	>100
5	GR-330	537034-17-6	**BML-210**	339.4	DMSO	>100	27.3927	5.9893	12.3709	12.8421	43.479	40.311
6	GR-340	183506-66-3	**Apicidin**	623.8	DMSO	0.1549	2.3047	0.3623	0.2266	0.3307	1.3011	0.7423
7	GR-323	38937-66-5	**Suberoyl bis-hydroxamic acid**	204.2	DMSO	15.952	50.1857	21.644	18.8828	24.5741	44.618	44.9859
8	GR-326	287383-59-9	**Scriptaid**	326.3	DMSO	0.6966	6.1234	1.0128	1.0517	1.1608	3.7929	3.2331
9	GR-327		**Nullscript**	298.3	DMSO	13.947	23.2412	10.64	15.3811	25.7078	47.756	54.5479
10	GR-345	2353-33-5	**5-Aza-2'-deoxycytidine**	228.2	DMSO	>33	>100	>100	>100	>100	>100	>100
11	GR-344	3690-10-6	**Zebularine**	228.2	DMSO	>100	>100	>100	>100	>100	>100	>100
12	ALX-270-288	149647-78-9	**SAHA**	264.3	DMSO	0.9251	4.2589	1.055	1.6499	1.7998	38.285	7.606
13	AC-1392	1453-82-3	**Isonicotinamide**	122.1	DMSO	>100	>100	>100	>100	>100	>100	>100
14	GR-350	200626-61-5	**ITSA-1**	292.1	DMSO	73.965	16.1077	12.936	19.176	18.9996	>33	30.0082
15	EI-320	1716-12-7	**Phenylbutyrate·Na**	186.2	DMSO	>100	>100	>100	>100	>100	>100	>100
16	EI-217	13492-01-8	**Tranylcypromine hemisulfate**	231.3	DMSO	>100	>100	>100	>100	>100	>100	>100
17	GR-352	99-66-1	**Valproic acid**	144.2	DMSO	66.5	>100	>100	>100	>100	>100	>100
18	GR-348	49843-98-3	**EX-527**	248.7	DMSO	>100	>100	>100	>100	>100	>100	>100
19	FR-104	501-36-0	**Resveratrol**	228.2	DMSO	10.298	>33	42.238	91.0518	43.9882	>100	91.3763
20	AC-1387	251456-60-7	**M-344**	307.4	DMSO	0.4521	1.2503	0.6951	3.0376	0.971	38.387	2.0154
21	KI-283	98-92-0	**Nicotinamide**	122.1	DMSO	>100	>100	>100	>100	>100	>100	>100
22	GR-346	96969-83-4	**BML-266**	478.5	DMSO	12.37	10.9201	13.812	>100	12.434	>100	65.8739
23	EI-271	10083-24-6	**Piceatannol**	244.2	DMSO	15.541	10.3103	7.208	26.3016	6.0924	27.606	26.0591
24	GR-353		**Fluoro-SAHA**	282.3	DMSO	1.8901	2.6082	2.1414	2.1693	2.1654	7.8716	4.375
25	GR-351	106132-78-9	**Valproic acid hydroxamate**	159.2	DMSO	>100	>100	>100	>100	>100	>100	>100
26	ALX-270-484	304896-28-4	**AGK2**	434.3	DMSO	5.0139	3.1706	2.4437	3.0294	3.4665	8.5679	3.557
27	AC-1388	1105698-15-4	**Salermide**	394.5	DMSO	26.247	27.298	24.184	33.6809	30.2191	>33	33.7859
28	ALX-270-344		**MC-1293**	284.3	DMSO	11.22	14.5847	7.4349	26.2408	12.3014	56.52	17.8472
29	ALX-270-381	16611-84-0	**Anacardic acid**	348.5	DMSO	>100	56.3218	45.678	37.6383	40.6905	62.281	42.5367
30	ALX-270-485	115687-05-3	**B2**	396.8	DMSO	78.064	45.7013	>100	48.8581	26.8853	>100	>33
31	ALX-270-473	935693-62-2	**BIX-01294·3HCl**	600.0	DMSO	1.7036	3.2179	3.497	3.9772	1.0137	3.6855	4.9786
32	ALX-270-411	778649-18-6	**Butyrolactone 3**	184.2	DMSO	>100	>100	>100	>100	>100	>100	>100
33	ALX-420-033	586976-24-1	**CTPB**	554.1	DMSO	>100	>100	97.61	64.6991	>100	>100	>100
34	ALX-270-379	151720-43-3	**Oxamflatin**	342.4	DMSO	0.6538	3.2368	0.5312	0.7172	1.3436	7.1415	2.5411
35	ALX-270-308	410536-97-9	**Sirtinol**	394.5	DMSO	28.276	34.2963	25.066	35.1046	40.3519	63.277	37.1015
36	G-430	129-46-4	**Suramin·6Na**	1429.2	DMSO	>100	>100	>100	>100	>100	>100	>100
37	GR-359		**BML-278**	391.5	DMSO	>100	>100	13.859	>100	>100	>100	>100
38	AC-1389	848354-66-5	**NCH-51**	390.6	DMSO	2.2291	5.5441	1.1233	3.3898	>100	17.327	3.1765
39	AC-1390	112522-64-2	**CI-994**	269.3	DMSO	6.7534	14.1748	3.3806	9.2777	>100	40.113	12.0091
40	AC-1391	3565-26-2	**NSC-3852**	174.2	DMSO	0.428	0.3433	0.1274	2.1294	0.5805	4.7059	0.5941
41	AC-1566		**Aminoresveratrol sulfate**	325.3	DMSO	24.088	57.4584	14.863	79.7562	7.7718	>100	51.3679
42	GR-361	1045792-66-2	**BML-281**	446.5	DMSO	0.4103	1.3408	0.4506	0.6317	0.3589	1.066	1.1366
43	FR-119	42206-94-0	**Triacetylresveratrol**	354.4	DMSO	4.4194	27.4777	15.844	30.2721	14.8603	>33	21.0472

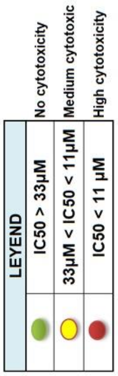

B

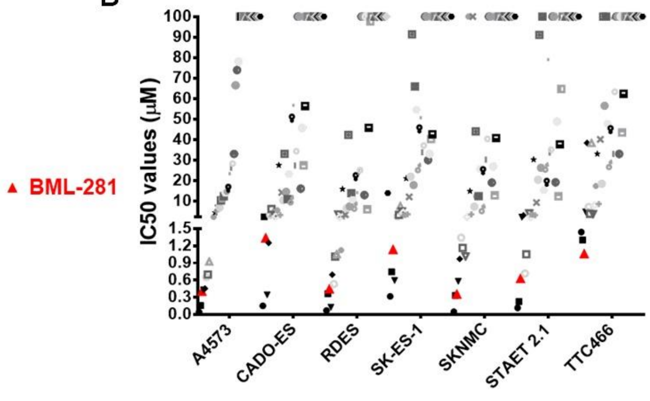

C

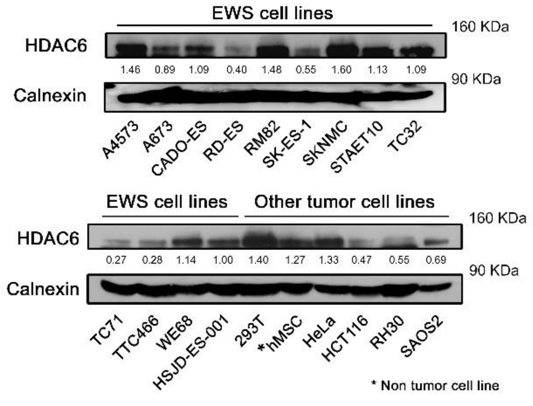

D

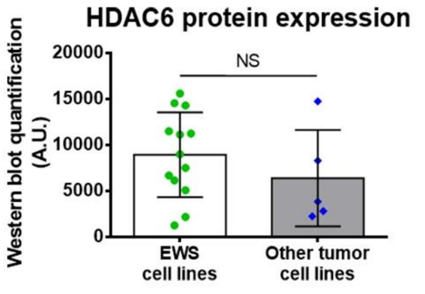

E

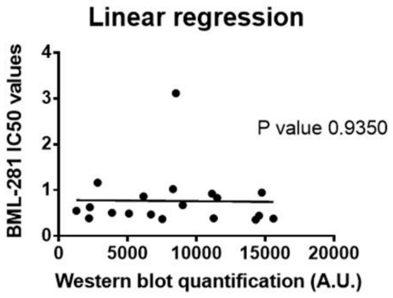

F

Cell lines	IC25 (μM)	IC75 (μM)	IC90 (μM)
A4573	0.227	0.560	0.874
A673	0.409	1.097	1.794
CADO-ES	0.622	1.377	2.068
RD-ES	0.326	0.742	1.133
RM82	0.312	0.626	0.888
SK-ES-1	0.600	1.319	2.101
SKNMC	0.252	0.574	0.864
STAET10	0.161	0.909	2.105
TC32	0.400	1.703	3.394
TC71	0.341	0.881	1.417
TTC466	0.244	0.615	0.983
WE68	0.229	0.594	0.958

Supplementary Fig.2

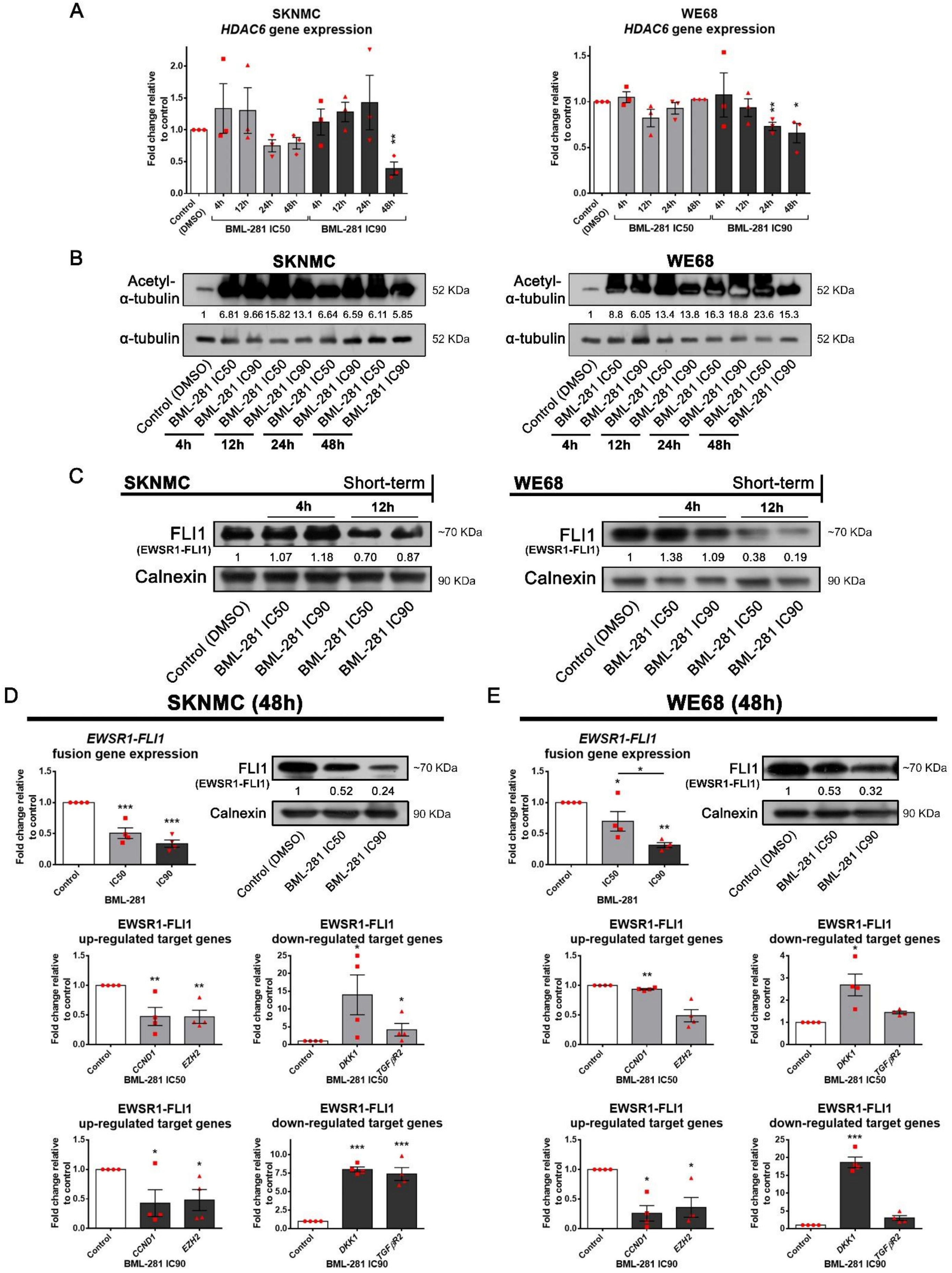

Supplementary Fig.3

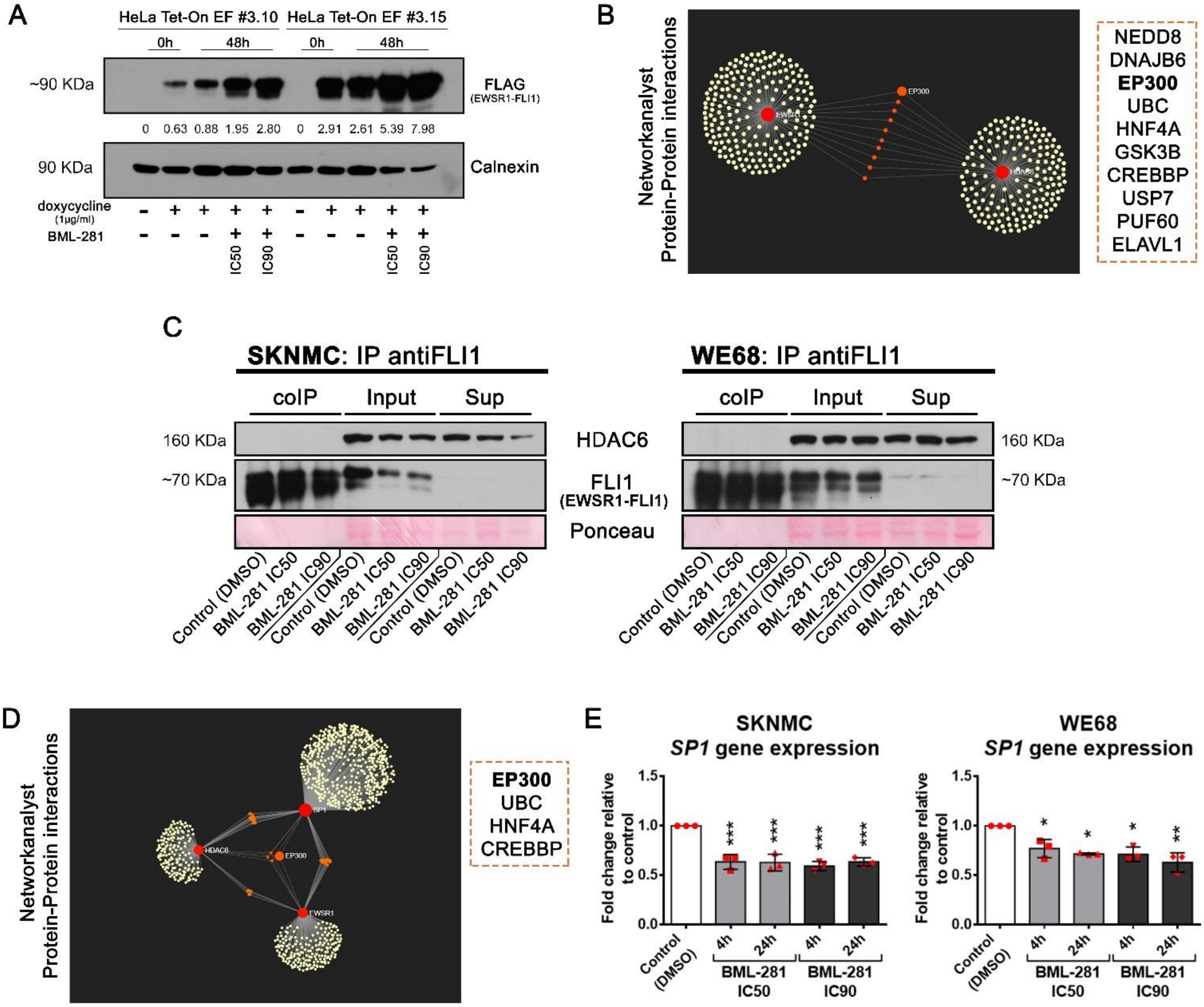

Supplementary Fig.4

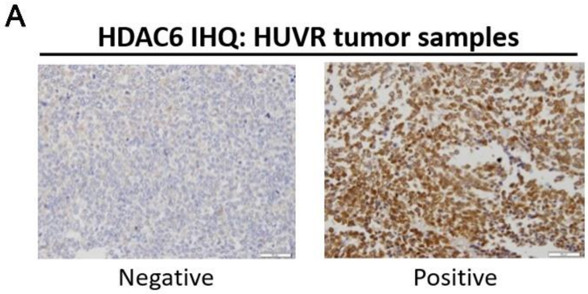

B

BML-281
DXR

	Combination Index (CI)	s.d.
A673	**0.723**	0.046
RM82	**0.686**	0.224
SKNMC	**0.804**	0.126
WE68	**0.513**	0.136
HSJD-ES-001	**0.730**	0.203

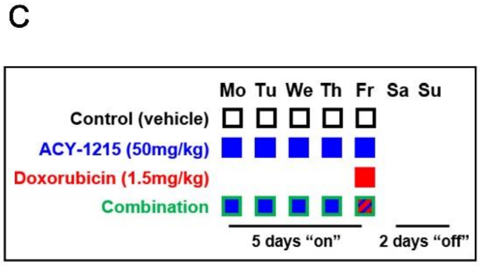

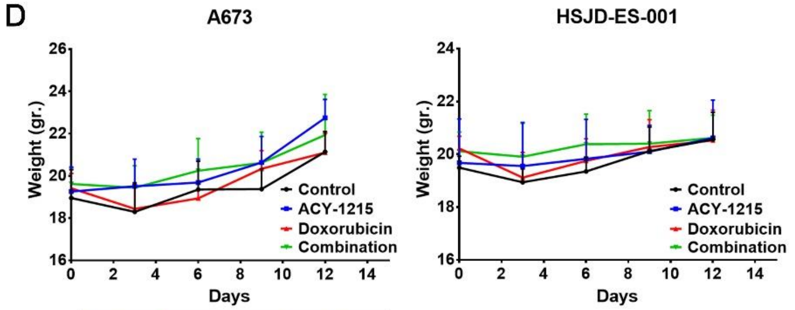

A673:

Dunnett's multiple comparisons test	P Value
Control vs. ACY-1215	0.4457
Control vs. Doxorubicin	0.9801
Control vs. Combination	0.4419

HSJD-ES-001:

Dunnett's multiple comparisons test	P Value
Control vs. ACY-1215	0.7391
Control vs. Doxorubicin	0.6942
Control vs. Combination	0.181

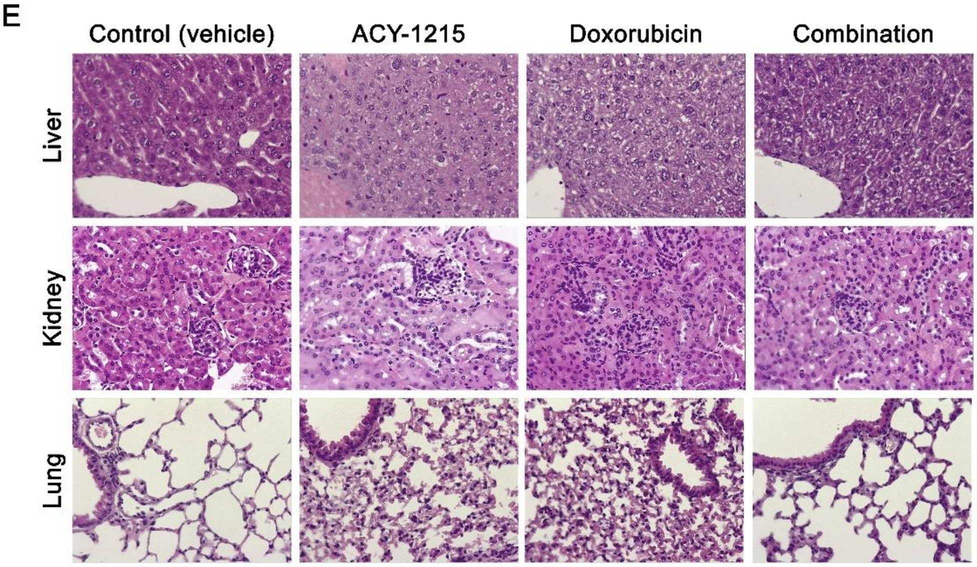

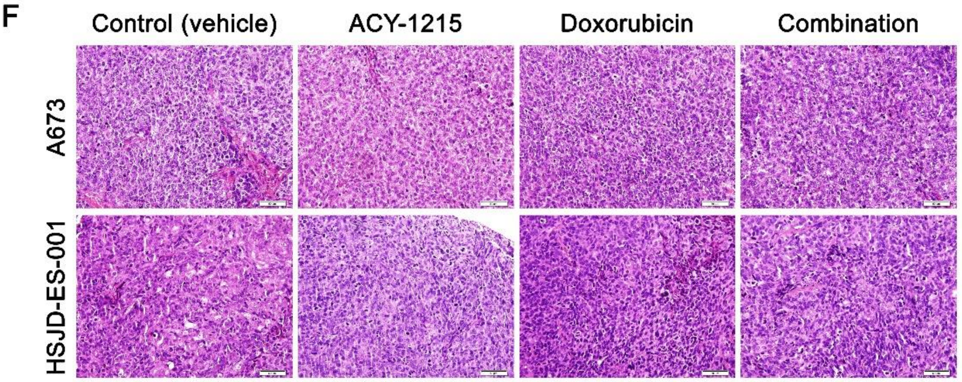

Supplementary Fig.1: (**A**) Summary table of the screened 43 epigenetic drug effects on the 50% inhibitory concentration (IC50) of seven EWS cell lines (Screen-Well® Epigenetics Library BML-2836, Enzo). (**B**) Schematic plot of the epigenetic drugs IC50 effectiveness (Screen-Well® Epigenetics Library BML-2836, Enzo) of seven EWS cell lines. (**C**) Immunoblotting evaluation of the HDAC6 protein expression level on cell lines (EWS, non-tumor, and other tumor). Numbers below blots represent densitometric quantification of bands, normalized to endogenous bands (calnexin). (**D**) Comparison of the average HDAC6 protein expression levels in EWS and other tumor cell lines. (**E**) Assessment of the relationship between the linear regression of the levels of HDAC6 protein expression and IC50 values of all cell lines studied treated with BML-281 treatment. (**F**) Assessment of IC25, IC75, and IC90 effects on 12 EWS cell lines exposed to BML-281 for 72 hours. *P < 0.05; **P < 0.01; ***P < 0.001.

Supplementary Fig.2: (**A**) RT-qPCR analyses of *HDAC6* mRNA levels in SKNMC and WE68 EWS cell lines treated with BML-281. (**B**) Immunoblotting evaluation of acetylated level of α-tubulin in SKNMC and WE68 cell lines treated with increasing concentrations of BML-281 (IC50 and IC90) at different time points. (**C**) Immunoblot evaluation of EWSR1-FLI1 protein expression levels in SKNMC and WE68 EWS cell lines treated with BML-281 for 4 or 12 hours, using IC50 or IC90 concentrations. (**D** and **E**) RT-qPCR and immunoblot assessment of mRNA and protein expression levels of EWSR1-FLI1 (upper panels), and EWSR1-FLI1 regulating target genes (lower panels) after 48 hours of BML-281 at IC50 and IC90 concentrations in SKNMC or WE68 cell line, respectively. Numbers below blots represent densitometric quantification of bands, normalized to endogenous bands and referred to their respective controls (DMSO band from the same time point). *P < 0.05; **P < 0.01; ***P < 0.001.

Supplementary Fig.3: (**A**) Immunoblot evaluation of EWSR1-FLI1 protein expression in two doxycycline-induced HeLa model clones treated with BML-281 at IC50 or IC90 concentrations for 48 hours. Numbers below blots represent densitometric quantification

of bands, normalized to endogenous bands (calnexin). (**B**) Protein-protein interaction predictions of candidates that connect HDAC6 and EWSR1 using the Global EnrichNetwork/networkanalyst. (**C**) Co-immunoprecipitation evaluation of HDAC6 physical interactions with the EWSR1-FLI1 protein with or without BML-281 treatment in SKNMC and WE68 cell lines. (**D**) Protein-protein interaction predictions of candidates that connect SP1, HDAC6, and EWSR1 using the Global EnrichNetwork/networkanalyst. (**E**) RT-qPCR assessment of *SP1* expression levels in SKNMC and WE68 EWS cell lines treated with BML-281 at IC50 or IC90 levels.

Supplementary Fig.4: (**A**) Immunohistochemical detection of HDAC6 expression in EWS patient tissue microarray (40× amplification). (**B**) Combination index (CI) of BML-281 and doxorubicin on proliferation inhibition in five EWS cell lines. (**C**) Schematic protocol of the *in vivo* experimental design and animal treatment. (**D**) Animal weight presentations of A673 and HSJD-ES-001 xenografts models, single-monitored, after single-agent or combination therapies. (**E**) H&E staining for morphological mice liver, kidney, and lung tissue toxicity assessment. (**F**) H&E staining for morphological xenograft mice tumor tissue assessment.

SUPPLEMENTAL TABLES

Supplementary Table 1: Summary of RNA-seq results for BML-281–treated SKNMC and WE68 cell lines at 4 hours or 24 hours after treatment.

Supplementary Table 2: Functional enrichment analysis based on gene ontology (GO) for BML-281–treated SKNMC and WE68 cell lines at 4 hours or 24 hours after treatment.

Supplementary Table 3: GSEA of rank-ordered gene sets (C2_MSigDB) in BML-281–treated SKNMC and WE68 signatures at 24 hours after treatment. Ranked gene set: NOM $P < 0.05$.

Supplementary Table 4: Clinicopathological features of the EWS patient tumor samples; Series1.

Supplementary Table 5: Clinicopathological features of the EWS patient tumor samples; Series2.

Supplementary Table 6: Materials or reagents used in the present study. Antibodies, TaqMan probes, and primers, as well as their respective sources and references are shown.
