# Supplementary material for: Selective inhibition of HDAC6 regulates expression of the oncogenic driver EWSR1-FLI1 through the EWSR1 promoter in Ewing sarcoma
Source: Oncogene. 2021 Aug 3;40(39):5843–53. doi: 10.1038/s41388-021-01974-4 (PMC8484017; doi:10.1038/s41388-021-01974-4)
Supplement: Supplementary file 5 — Supplementary Table 4 [file 41388_2021_1974_MOESM5_ESM.docx]

**Supplementary Table 4:** Clinicopathological features of the EWS patient tumor samples; Series1

|  |  | **Patient series** |  |
| --- | --- | --- | --- |
| **Samples** |  | Paraffin tumors |  |
| **Number of patients** |  | 52 |  |
| **Age in years (range)** |  | 2–45 years |  |
| **Samples type** |  |  |  |
| Primary tumors |  | 41 |  |
| Recurrent tumors |  | 17 |  |
| No recurrent tumors |  | 24 |  |
| No data available |  | 11 |  |
| **Location** |  |  |  |
| Bone |  | 27 |  |
| Soft tissue |  | 17 |  |
| No data available |  | 8 |  |
|  |  |  |  |
|  |  |  |  |
