# Supplementary material for: Selective inhibition of HDAC6 regulates expression of the oncogenic driver EWSR1-FLI1 through the EWSR1 promoter in Ewing sarcoma
Source: Oncogene. 2021 Aug 3;40(39):5843–53. doi: 10.1038/s41388-021-01974-4 (PMC8484017; doi:10.1038/s41388-021-01974-4)
Supplement: Supplementary file 7 — Supplementary Table 6 [file 41388_2021_1974_MOESM7_ESM.docx]

**Supplementary Table 6. Materials or reagents used in the present study.** Antibodies, TaqMan probes, and primers, as well as their respective sources and references are shown.

| **Antibodies** | | |
| --- | --- | --- |
| **Protein anti-** | **Brand** | **References** |
| Calnexin (E-10) | Santa Cruz | SC-46669 |
| FLAG-M2 | SIGMA | F3165 |
| FLI1 | MyBiosource | MBS300723 |
| H4K12Ac | Millipore | 06-1352-I |
| HDAC6 | Cell Signaling | 7558 |
| IgG | Santa Cruz | SC-2025 |
| Ki67 | Roche | clone 30-9 |
| Laminin | Abcam | ab26300 |
| Lysine Ac | Arigo | ARG20523 |
| P300 (C20) | Santa Cruz | SC-585 |
| SP1 | Millipore | 07-645 |
| TH4 | Cell Signaling | 13919S |
| α-Tubulin | Cell Signaling | 2144S |
| α-TubulinAc | Cell Signaling | 12152S |
| Calnexin (E-10) | Santa Cruz | SC-46669 |
| **Taqman probes used for qRT-PCR** | | |
| **Genes** | **Brand** | **References** |
| *CCND1* | Thermo Fisher | Hs00765553_m1 |
| *DKK1* | Thermo Fisher | Hs00183740_m1 |
| *EWSR1 (endogenous)* | Thermo Fisher | Hs01580530_gH |
| *EWSR1-FLI1* | Thermo Fisher | Hs03024497_ft |
| *EZH2* | Thermo Fisher | Hs00544830_m1 |
| *HDAC6* | Thermo Fisher | Hs00195869_m1 |
| *SP1* | Thermo Fisher | Hs00916521_m1 |
| *TGFβR2* | Thermo Fisher | Hs00234253_m1 |
| *TPT1* | Thermo Fisher | Hs02621289_g1 |
| miR-Let-7a | Thermo Fisher | 000377 |
| miR-Let-7b | Thermo Fisher | 000378 |
| miR-Let-7c | Thermo Fisher | 000379 |
| RNU44 | Thermo Fisher | 001094 |
| RNU48 | Thermo Fisher | 001006 |
| **Primers for ChIP** | | |
| **Genes** | **Sequence** | |
| *EWSR1* activating region_Fw | CAAACAGCCTAGTCTCGTGC | |
| *EWSR1* activating region_Rv | GACACTCGGGCCAAAATAGC | |
| Control negative region_Fw | AAGTCCCACATCTTGAGCCA | |
| Control negative region_Rv | GAGGCTTACAGTGGATGGGT | |
