# Supplementary material for: Selective inhibition of HDAC6 regulates expression of the oncogenic driver EWSR1-FLI1 through the EWSR1 promoter in Ewing sarcoma
Source: Oncogene. 2021 Aug 3;40(39):5843–53. doi: 10.1038/s41388-021-01974-4 (PMC8484017; doi:10.1038/s41388-021-01974-4)
Supplement: Supplementary file 8 — Supplemental Materials and Methods [file 41388_2021_1974_MOESM8_ESM.docx]

**supplementary MATERIALS AND METHODS**

**1) Cultured cell lines and pharmaceutical compounds.** The cell lines A4573, A673, CADO-ES, RD-ES, RM82, SK-ES-1, SKNMC, STAET10, TC32, TC71, TTC466, and WE68 were obtained from ATCC and the EuroBoNet cell line panel ^9^. EWS cell lines were grown on 0.1% gelatin-coated plates in RPMI 10% FBS except for A673 (DMEM 10%), SK-ES-1 (McCoy's 15%), SKNMC (EMEM 10%), and RDES (RPMI 15%). The HSJD-ES-001 (an EWS patient-derived cell line, developed in Sant Joan de Déu Hospital, Barcelona ^4^), RH30 (rhabdomyosarcoma cell), and hMSC cells lines were grown in RPMI 10% FBS. HCT116 (colorectal carcinoma cell line), SAOS-2 (osteosarcoma cell line), HEK293T, and HeLa (cervix cancer) cells were grown in DMEM 10%. All media were supplemented with 100 U/ml penicillin and 100 µg/ml streptomycin. Cells were maintained in 37°C incubators, in an atmosphere of 5% CO_2_. All cells were free of mycoplasma, as screened with the MycoAlert® Mycoplasma Detection Kit (Lonza). Cell lines were maintained and regularly checked and characterized by polymorphism analysis (conducted by CLS Cell Lines Service Company).

BML-281 (#BML-GR361) and ACY-1215 (#21531) were purchased from Enzo Life Science Inc. (USA) and Cayman Chemical (USA), respectively. Stock solutions of BML-281 was prepared in dimethyl sulfoxide (DMSO) and diluted to final concentration in the culture medium 1:1000 (v/v). The SCREEN-WELL® Epigenetics library (BML-2836) was purchased from Enzo Life Science Inc. (USA) and stored at −80°C until use.

**2) Cell viability assays.** BML-281 was added to complete growth medium at concentrations ranging from 0.01 to 100 µM to calculate the IC values in monotherapy. After 72 hours, cells were subjected to an ATP-lite assay (PerkinElmer, Waltham, MA, USA), and inhibitory concentrations were calculated using OriginPro 9.0.0 (OriginLab). Combination index (CI) values were based on the mean growth inhibitions of BML-281 and Doxorubicin in monotherapy and in combination. CI values were calculated according to the Chou-Talalay method ^3^. Synergy levels can be broadly divided into: <0.1, very strong synergism; 0.1–0.9, synergism (ranging from strong synergism to slight synergism); 0.9–1.1, nearly additive to additive; and >1.1, antagonism.

**3) Protein extraction and Western blot.** Proteins were extracted from cell lines in RIPA buffer (150 mM NaCl, 1% (v/v) NP40, 50 mM Tris-HCl pH 8.0, 0.1% (v/v) SDS, 1 mM EDTA, and 0.5% (w/v) deoxycholate) supplemented with 10 mM NaF and 2 mM NaOv protease inhibitors. Immunoblotting was performed using the antibodies listed in Supplementary Table 6. Protein bands were visualized using the Clarity Western ECL Substrate chemiluminescence detection kit (Bio-Rad, #170-5060). ImageJ 1.52p software was used for densitometric quantifications.

**4) Flow cytometry analyses.** Cell flow cytometry analyses were conducted to evaluate apoptosis (48 hours) after treatment. These assays were performed as previously described ^4^. Flow cytometry data were processed and analyzed with FlowJo software (Tree Star).

**5) Clonogenic assay (3D culture-soft agar)**. Cells were treated for 24 hours with BML-281 at different concentrations (IC50 and IC90). Cells were then washed, trypsinized, and counted, Cells (5 × 10^4^/per well) were either treated or not (as control cells) and plated onto soft agar (1.5% in completed medium without treatment) in 6-well plates and incubated for 21 days to allow colony formation. Colonies were fixed and then stained with a mixture of 6.0% glutaraldehyde and 0.5% crystal violet solution. Colonies were counted manually after drying.

**6) Nucleus and cytoplasm extraction.** To isolate subcellular fractions, cells were lysed on ice in 800 µl of 10 mM HEPES pH 7.9 buffer containing 10 mM KCl, 0.1 mM EDTA, and 0.1 mM EGTA and incubated on ice for 15 min; tubes were then centrifuged for 2 min at 15,700*g* at 4°C. Supernatant (cytoplasmic) fractions were transferred to a clean, pre-chilled tube and placed on ice until use or frozen for storage at –80°C. Nuclear fractions in the pellets were resuspended in 75 µl of 20 mM HEPES pH 7.9 buffer containing 0.4 M NaCl, 1 mM EDTA, 1 mM EGTA, and the protease inhibitors cocktail solution. After 15 min of incubation on ice, tubes were centrifuged for 5 min at 15,700*g* at 4°C. Supernatants (nuclear fractions) were transferred to a clean, pre-chilled tubes and placed on ice until use or frozen for storage at –80°C.

**7) mRNA expression analysis.** Expression levels of selected genes were analyzed by RT-qPCR. RNA was isolated from cell lines using the miRVana miRNA Isolation Kit (Ambion; Life Technologies, USA). The quantity and quality of the total RNA was determined with the Nanodrop ND-2000 Spectrophotometer (Thermo Scientific). Prior reverse transcription was performed using TaqMan Reverse Transcription Kit (Applied Biosystems; Life Technologies) in GeneAmp PCR 9700 thermocycler, and RT-qPCR amplification, with the TaqMan Universal PCR Master Mix (Applied Biosystems) to measure expression of the miR-Let-7 family (miR-let-7a/b/c), using RNU44 and RNU48 as endogenous controls. Prior reverse transcription was performed using the TaqMan MicroRNA Reverse Transcription Kit (Applied Biosystems). All RT-qPCR measurements were obtained in a 7900HT Fast Real Time PCR System with ExpressionSuite Software v1.0 (Applied Biosystems). TaqMan probes are listed in Supplementary Table 6.

**8) Chromatin immunoprecipitation (ChIP).** EWS cells were fixed with 1% formaldehyde at room temperature for 10 min, followed by 5 min quenching with 0.125 M glycine, and then washed once in ice-cold PBS. Pellets were resuspended in lysis buffer (0.1% SDS, 0.1 M NaCl, 1% Triton X-100, 1 mM EDTA, 20 mM Tris pH 8, and 1 mg/ml protease inhibitors) and sonicated with a Bioruptor until the crosslinked chromatin was sheared, with an average DNA fragment length of 0.5 kbp. After centrifugation (30 min at 15,700 *g*), chromatin preparations were precleared by incubation with 40 μl of protein A agarose/salmon sperm DNA, 50% gel slurry (Millipore) for 2 hours at 4˚C under rotation. Protein A agarose was removed by centrifugation, and the precleared chromatin was immunoprecipitated by incubation with 5 μg anti-SP1 or anti-p300 antibodies and 50 μl protein A agarose overnight at 4˚C. Washed pellets were eluted with 120 μl of a solution containing 1% SDS, 0.1 M NaHCO_3_. Eluted pellets were de-crosslinked at 65˚C overnight and purified in 50 μl Tris-EDTA buffer using the QIAquick PCR Purification Kit (Qiagen). Differences in the DNA content from every immunoprecipitation assay were determined by real-time PCR using the ABI 7700 sequence detection system and SYBR Green master mix protocol (Applied Biosystems). Primers used in this study are listed in Supplementary Table 6. The reported data represent real-time PCR values normalized to input DNA and expressed as percentage (%) of bound/input signals.

**9) Proteins co-immunoprecipitation.** Whole protein extracts (250 µg) in NP40 buffer (150 mM NaCl, 20 mM Tris pH 8.0, 1 mM DTT, 0.5% NP40) were incubated with 15 µl of protein A-dynabeads (#10001D, Invitrogen) coupled to 2 µg of polyclonal anti-FLI1 (MyBiosource, #MBS300723) or anti-SP1 (Millipore, #07-645), for 3 hours at 4ªC. After magnetic immunoprecipitation and washes, immunoprecipitates were resolved in a 10% polyacrylamide SDS-PAGE gel, transferred, and blotted as described above.

**10) Immunohistochemistry.** Representative sections were incubated with primary antibodies overnight at 4°C. The antibody used were HDAC6 1:600 (Cell Signaling #7558), anti-Ki-67 1:100 (clone 30-9, Roche), and anti-FLI1 1:100 (MyBiosource, #MBS300723). Peroxidase-labelled secondary antibodies and 3, 3′-diaminobenzidine were applied to develop immunoreactivity, according to manufacturer’s protocol (EnVision; Dako, Glostrup, Denmark). The histopathological study using hematoxylin &eosin (H&E) staining and HDAC6 staining was evaluated independently by two pathologists (MJR and EDA). The evaluation was blinded. Ki67 labelling was quantified by ImageJ 1.52p software.

**11) Statistics.** Differences between control and treatment conditions were evaluated using Mann–Whitney U-test for two groups, and one-way analysis of variance test for more than two groups followed by post hoc Tukey’s multiple comparisons. The disease-free survival (DFS) time was analyzed using the Kaplan–Meier estimator and the Wilcoxon test. Analyses were performed using the Prism 6 software (GraphPad). Normal distribution of data was assessed before application of parametric tests. The variance was similar between groups that were being statistically compared. The data met the assumptions of the tests. For all analyses, P values of ≤ 0.05 were considered statistically significant. The average of at least three biological replicates performed in three technical replicates with SEM is presented in all experiments. No data were excluded from the analyses.

For cell experiments study, samples were no randomized and sample size was determined to be adequate based on the magnitude and consistency of measurable differences between groups, usually the number is three or more. For xenograft mice experiment, no statistical methods were used to predetermine sample size, which was determined based on previous experimental observations.

**12) Next-generation transcriptome sequencing (RNA-Seq) for gene expression analysis.** Total RNA from 18 samples was sequenced in a NextSeq 500 sequencer (Illumina, CA, USA) producing 23,830,047 raw 75×2 nt paired-end reads on average. Sequences were processed using miARma-Seq pipeline^1^. Briefly, quality filtered reads were aligned using HISAT2^5^, resulting in a 90.13% of properly aligned reads that were summarize into gene expression values using feature Counts^6^. Differential expression analysis was done using the edgeR package^8^, generating DEG between time points, using both tissues at 4h and 24h (6 replicates per time) versus control conditions (6 replicates). Next, a Gene Set Enrichment Analysis (GSEA)^13^ was performed to understand the resulting expression profile considering DEG (FDR<0.05 and logfc>|1.5|). Normalized enrichment scores NES were calculated between DEG against a curated dataset of MSigDB, called C2 CGP, which comprises 3358 gene sets representing expression signatures of Genetic and Chemical Perturbations (see supplementary information for further details).

**Data Analysis.** miARma-Seq pipeline was used to analyse transcriptomic samples ^1^. Firstly, raw data were evaluated using FastQC software to assess the quality of the reads ^2^. Subsequently, after sample filtering, a mean of 47% in GC content and an average of 19,087,408 reads per sample were obtained (Table A).

miARma-Seq then aligns all quality filtered sequences using HISAT2 ^5^, which resulted in a 90.13% of properly aligned reads. With this aim, *Homo sapiens* Gencode version v26 genome-build: GRCh38 was used. The featureCounts software ^6^ was then used to assign sequence reads to genes by using reference gene annotation obtained from Gencode from the same assembly and genome build (Table B).

**Differential expression.** Differential expression analysis was performed using edgeR package ^8^. Low-level expressed genes were removed, and the remaining genes were normalized by the trimmed mean of M-values (TMM) method ^12^. Reads per kilo base per million mapped reads (RPKM), counts per million (CPM), and log2-counts per million (log-CPM) were calculated per gene on each sample ^8^. Principal component analysis (PCA) and hierarchical clustering of normalized samples were used to get a general overview on the similarity of RNA-sequencing samples ^10, 11^.

Differentially-expressed genes (DEG) were calculated between time points, using tissue at 4 hours or 24 hours (with 6 replicates per time) versus control conditions (6 replicates); for this, a false discovery rate (FDR) of <0.05, and log2 FC were used to evaluate the significance and changes in expression, respectively, of a gene between both types of samples.

**Enrichment analysis.** In order to identify the effects of differential gene expression, a functional enrichment study was carried out using the *clusterProfiler* Bioconductor package ^14^. DEGs were compared against all expressed genes in the RNA-seq assay, and gene ontology (GO) terms were obtained from the Bioconductor human database and associated to Entrez gene identifiers in an *orgDB* R object, using the *AnnotationForge* package with *clusterProfiler*.

GO enrichment analyses were evaluated for biological process, molecular function, and cellular complex ontology terms. KEGG pathway enrichment was also calculated from human gene names previously acquired from the Gencode annotation.

**GSEA preranked calculation.** Gene set enrichment analysis (GSEA; Subramanian et al., 2005) was used to understand the expression profiles that results from this study. The most similar signature was found in the Molecular Signatures Database (MSIgDB), which includes a collections of annotated gene sets to be used with GSEA to help represent a wider range of biological processes and diseases from transcriptomic analyses ^7^.

All differentially expressed genes (DEG) were considered and sorted, to carry out a GSEA preranked analysis. GSEA identified gene sets from MSigDB and compared them with genes that have a greater impact as compared to all genes, with altered expression in own conditions. Normalized enrichment scores NES were calculated between differentially expressed genes obtained in this study against a curated dataset of MSigDB, called C2 CGP, which comprises 3358 gene sets representing expression signatures of the genetic and chemical perturbations categories.

**Table A. Summary of pre-fastqc results using the miARma software**

| Name | Number of reads | % GC Content | Read Length |
| --- | --- | --- | --- |
| SKNMC_0h_rep1_R1 | 17803824 | 48% | 75 |
| SKNMC_0h_rep1_R2 | 17803824 | 48% | 75 |
| SKNMC_0h_rep2_R1 | 22471451 | 47% | 75 |
| SKNMC_0h_rep2_R2 | 22471451 | 48% | 75 |
| SKNMC_0h_rep3_R1 | 17044401 | 48% | 75 |
| SKNMC_0h_rep3_R2 | 17044401 | 49% | 75 |
| SKNMC_4h_rep1_R1 | 19323371 | 47% | 75 |
| SKNMC_4h_rep1_R2 | 19323371 | 47% | 75 |
| SKNMC_4h_rep2_R1 | 16324412 | 49% | 75 |
| SKNMC_4h_rep2_R2 | 16324412 | 49% | 75 |
| SKNMC_4h_rep3_R1 | 20551582 | 47% | 75 |
| SKNMC_4h_rep3_R2 | 20551582 | 48% | 75 |
| SKNMC_24h_rep1_R1 | 16339928 | 48% | 76 |
| SKNMC_24h_rep1_R2 | 16339928 | 49% | 76 |
| SKNMC_24h_rep2_R1 | 23950269 | 47% | 76 |
| SKNMC_24h_rep2_R2 | 23950269 | 47% | 76 |
| SKNMC_24h_rep3_R1 | 16181849 | 48% | 76 |
| SKNMC_24h_rep3_R2 | 16181849 | 48% | 76 |
| WE_0h_rep1_R1 | 18648636 | 46% | 75 |
| WE_0h_rep1_R2 | 18648636 | 46% | 75 |
| WE_0h_rep2_R1 | 18839842 | 47% | 75 |
| WE_0h_rep2_R2 | 18839842 | 47% | 75 |
| WE_0h_rep3_R1 | 17110025 | 48% | 75 |
| WE_0h_rep3_R2 | 17110025 | 48% | 75 |
| WE_4h_rep1_R1 | 24686958 | 49% | 76 |
| WE_4h_rep1_R2 | 24686958 | 50% | 76 |
| WE_4h_rep2_R1 | 16516498 | 47% | 76 |
| WE_4h_rep2_R2 | 16516498 | 47% | 76 |
| WE_4h_rep3_R1 | 20135263 | 47% | 76 |
| WE_4h_rep3_R2 | 20135263 | 47% | 76 |
| WE_24h_rep1_R1 | 18537879 | 47% | 75 |
| WE_24h_rep1_R2 | 18537879 | 47% | 75 |
| WE_24h_rep2_R1 | 20300497 | 45% | 75 |
| WE_24h_rep2_R2 | 20300497 | 45% | 75 |
| WE_24h_rep3_R1 | 18806665 | 46% | 75 |
| WE_24h_rep3_R2 | 18806665 | 46% | 75 |

**Table B. Summary of assigned reads using the miARma software**

| Name | Processed Reads | Assigned reads | Number of identified entities |
| --- | --- | --- | --- |
| SKNMC_0h_rep1 | 21193730 | 12796174 (60.4%) | 25953 |
| SKNMC_0h_rep2 | 28641426 | 15513032 (54.2%) | 26893 |
| SKNMC_0h_rep3 | 20695947 | 11952102 (57.8%) | 25430 |
| SKNMC_4h_rep1 | 26411169 | 12685293 (48.0%) | 26582 |
| SKNMC_4h_rep2 | 20197264 | 10990535 (54.4%) | 25808 |
| SKNMC_4h_rep3 | 26439645 | 14021266 (53.0%) | 26503 |
| SKNMC_24h_rep1 | 20094634 | 10847690 (54.0%) | 27583 |
| SKNMC_24h_rep2 | 28347693 | 17261118 (60.9%) | 26811 |
| SKNMC_24h_rep3 | 19876898 | 10704972 (53.9%) | 26855 |
| WE_0h_rep1 | 22584188 | 12073842 (53.5%) | 25914 |
| WE_0h_rep2 | 23249043 | 11510714 (49.5%) | 25708 |
| WE_0h_rep3 | 20739550 | 10959828 (52.8%) | 24884 |
| WE_4h_rep1 | 31552507 | 15310464 (48.5%) | 26872 |
| WE_4h_rep2 | 19969615 | 10507670 (52.6%) | 24970 |
| WE_4h_rep3 | 28289543 | 11954047 (42.3%) | 25497 |
| WE_24h_rep1 | 24457899 | 11939695 (48.8%) | 26425 |
| WE_24h_rep2 | 23973260 | 12047066 (50.3%) | 26706 |
| WE_24h_rep3 | 22226842 | 11081049 (49.9%) | 26352 |

**References**

1 Andres-Leon E, Nunez-Torres R, Rojas AM. miARma-Seq: a comprehensive tool for miRNA, mRNA and circRNA analysis. Sci Rep 2016; 6: 25749.

2 Andrews_S. FastQC: a quality control tool for high throughput sequence data. Available online at: <http://www.bioinformatics.babraham.ac.uk/projects/fastqc> 2010.

3 Chou TC, Motzer RJ, Tong Y, Bosl GJ. Computerized quantitation of synergism and antagonism of taxol, topotecan, and cisplatin against human teratocarcinoma cell growth: a rational approach to clinical protocol design. J Natl Cancer Inst 1994; 86: 1517-1524.

4 Garcia-Dominguez DJ, Hontecillas-Prieto L, Rodriguez-Nunez P, Pascual-Pasto G, Vila-Ubach M, Garcia-Mejias R *et al*. The combination of epigenetic drugs SAHA and HCI-2509 synergistically inhibits EWS-FLI1 and tumor growth in Ewing sarcoma. Oncotarget 2018; 9: 31397-31410.

5 Kim D, Langmead B, Salzberg SL. HISAT: a fast spliced aligner with low memory requirements. Nat Methods 2015; 12: 357-360.

6 Liao Y, Smyth GK, Shi W. featureCounts: an efficient general purpose program for assigning sequence reads to genomic features. Bioinformatics 2014; 30: 923-930.

7 Liberzon A, Birger C, Thorvaldsdottir H, Ghandi M, Mesirov JP, Tamayo P. The Molecular Signatures Database (MSigDB) hallmark gene set collection. Cell systems 2015; 1: 417-425.

8 Nikolayeva O, Robinson MD. edgeR for differential RNA-seq and ChIP-seq analysis: an application to stem cell biology. Methods Mol Biol 2014; 1150: 45-79.

9 Ottaviano L, Schaefer KL, Gajewski M, Huckenbeck W, Baldus S, Rogel U *et al*. Molecular characterization of commonly used cell lines for bone tumor research: a trans-European EuroBoNet effort. Genes Chromosomes Cancer 2010; 49: 40-51.

10 Reeb PD, Bramardi SJ, Steibel JP. Assessing Dissimilarity Measures for Sample-Based Hierarchical Clustering of RNA Sequencing Data Using Plasmode Datasets. PLoS One 2015; 10: e0132310.

11 Ritchie ME, Phipson B, Wu D, Hu Y, Law CW, Shi W *et al*. limma powers differential expression analyses for RNA-sequencing and microarray studies. Nucleic Acids Res 2015; 43: e47.

12 Robinson MD, Oshlack A. A scaling normalization method for differential expression analysis of RNA-seq data. Genome Biol 2010; 11: R25.

13 Subramanian A, Tamayo P, Mootha VK, Mukherjee S, Ebert BL, Gillette MA *et al*. Gene set enrichment analysis: a knowledge-based approach for interpreting genome-wide expression profiles. Proc Natl Acad Sci U S A 2005; 102: 15545-15550.

14 Yu G, Wang LG, Han Y, He QY. clusterProfiler: an R package for comparing biological themes among gene clusters. OMICS 2012; 16: 284-287.
